# Early life starvation and Hedgehog-related signaling activate innate immunity downstream of *daf-18/PTEN* and *lin-35/Rb* causing developmental pathology in adult *C. elegans*

**DOI:** 10.1371/journal.pgen.1011641

**Published:** 2025-12-03

**Authors:** Ivan B. Falsztyn, James M. Jordan, Jingxian Chen, Winnie Zhao, Rojin Chitrakar, Aaron W. Reinke, L. Ryan Baugh

**Affiliations:** 1 Department of Biology, Duke University Box, Durham, North Carolina, United States of America; 2 University Program in Genetics and Genomics, Duke School of Medicine, Durham, North Caroline, United States of America; 3 Department of Molecular Genetics, University of Toronto, Toronto, Ontario, Canada; Centre National de la Recherche Scientifique, FRANCE

## Abstract

Early life experiences such as malnutrition can affect development and adult disease risk, but the molecular basis of such protracted effects is poorly understood. In the nematode *C. elegans,* extended starvation during the first larval stage causes the development of germline tumors and other abnormalities in the adult gonad, limiting reproductive success. Insulin/IGF signaling (IIS) acts through WNT signaling and lipid metabolism to promote starvation-induced gonad abnormalities, but IIS-independent modifiers have not been identified. The tumor suppressor *daf-18/PTEN* inhibits IIS to suppress starvation-induced abnormalities, but we show that it also acts independently of IIS via *lin-35/Rb*, another tumor suppressor, to suppress such abnormalities. We found that *lin-35/Rb* and the rest of the DREAM complex repress transcription of the Hedgehog (Hh) signaling homologs *ptr-23/PTCH-related*, *wrt-1/Hh-like*, and *wrt-10/Hh-like*, which promote starvation-induced abnormalities. These Hh-related genes transcriptionally activate several genes associated with innate immunity in adults, which also promote starvation-induced gonad abnormalities. Surprisingly, we found that in addition to causing developmental abnormalities, early-life starvation induces an innate immune response later in life, leading to increased resistance to bacterial and intracellular pathogens. This work identifies a critical tumor-suppressor function of *daf-18/PTEN* independent of IIS, and it defines a regulatory network, including *lin-35/Rb* and DREAM, Hh-related signaling, and innate immunity pathways, that affects development of tumors and other developmental abnormalities resulting from early life starvation. By revealing that early-life starvation increases immunity later in life, this work suggests a fitness tradeoff between pathogen resistance and developmental robustness.

## Author summary

Early life malnutrition can promote adult disease, including the risk of developing cancer. We previously found that the roundworm *Caenorhabditis elegans* develops tumors in well-fed adults following early life starvation, reflecting a breakdown of developmental fidelity. This powerful model system provides an excellent opportunity to identify molecular mechanisms that mediate the effects of early life starvation on development and adult physiology. Reducing insulin-like/IGF signaling during recovery from starvation suppresses tumor formation, but other regulatory pathways have not been identified. Here we show that a pair of important tumor suppressor genes, *daf-18/PTEN* and *lin-35/Rb*, function independently of insulin/IGF signaling to suppress starvation-induced abnormalities. *lin-35/Rb* represses genes involved in Hedgehog signaling, a cancer-causing pathway, which promote starvation-induced abnormalities. Hedgehog signaling activates an immune response, which, surprisingly, contributes to formation of tumors but renders adult worms resistant to pathogens. Adult activation of this immune response by early life starvation suggests an evolutionary fitness tradeoff between pathogen resistance and developmental fidelity.

## Introduction

Fetal malnutrition is associated with increased risk of type II diabetes, cancer, and obesity in adulthood [1–3]. Given confounding genetic and environmental factors in humans, tractable animal models are necessary to identify the molecular basis of such developmental origins of adult health and disease. The nematode *Caenorhabditis elegans* remains in a developmentally arrested state in the first larval stage (L1) after hatching without food (“L1 arrest” or “L1 diapause”) [4,5]. Starved larvae are able to survive L1 arrest for a couple of weeks and resume development upon feeding. However, a significant fraction of individuals subjected to extended L1 arrest (*e.g.*, 8 days) develop proximal germline tumors, differentiated uterine masses, and other reproductive abnormalities on the first day of adulthood following recovery in replete conditions [6–9]. With its short generation time, ease of manipulation, and powerful genetic toolkit, *C. elegans* L1 arrest and recovery provides a powerful system to study the effects of early life starvation on adult health and disease.

Insulin/insulin-like growth factor (IGF) signaling (IIS) regulates developmental plasticity, aging, metabolism, and starvation resistance [4,5,10]. Disruption of the sole known insulin/IGF receptor DAF-2/InsR increases survival during L1 arrest [11–13], and *daf-2/InsR* RNAi during larval development after L1 arrest suppresses starvation-induced gonad abnormalities, including germline tumors and uterine masses [6–9]. DAF-2/InsR signals through the phosphoinositide 3-kinase (PI3K) pathway by activating AGE-1/PI3K, which phosphorylates phosphatidylinositol-4, 5-bisphosphate (PIP2) to produce phosphatidylinositol-3, 4, 5-triphosphate (PIP3) [10,14,15]. PIP3 activates AKT and PDK kinases, which antagonize the forkhead box O transcription factor DAF-16/FoxO [16]. Disruption of *daf-2/InsR* increases DAF-16/FoxO activity, and *daf-16* is required for starvation resistance and suppression of starvation-induced gonad abnormalities with *daf-2* RNAi [6,12]. WNT signaling and lipid metabolism operate downstream of IIS to promote starvation-induced abnormalities [7,8], but pathways affecting such abnormalities independently of IIS have not been identified.

The tumor suppressor phosphatase and tensin (PTEN) homolog DAF-18/PTEN inhibits PI3K/IIS by dephosphorylating PIP3 to produce PIP2, counteracting AGE-1/PI3K activity [17]. Loss of *daf-18/PTEN* constitutively activates IIS, inhibiting *daf-16/FoxO*, thereby reducing starvation resistance and enhancing development of starvation-induced gonad abnormalities [8,18,19]. However, disruption of *daf-2/InsR* does not completely suppress starvation-induced abnormalities, and loss of *daf-16/FoxO* does not increase penetrance of such abnormalities, though loss of *daf-18/PTEN* does. These observations suggest that DAF-18 functions independently of IIS to suppress development of germline tumors following early life starvation. In addition to being a lipid phosphatase, PTEN is a protein phosphatase [20], supporting the possibility that DAF-18/PTEN tumor-suppressor function may extend beyond dephosphorylation of PIP3 and inhibition of PI3K/IIS [21].

Here we show that *daf-18/PTEN* functions independently of IIS during larval development after L1 arrest to suppress starvation-induced gonad abnormalities. *daf-18* does so by acting through another important tumor suppressor, *lin-35/Rb*. We show that *lin-35* and the rest of the DREAM complex repress transcription of Hedgehog signaling homologs (Hh-related) to mediate suppression of abnormalities. Bulk RNA-seq and reporter gene analysis in adults subjected to extended L1 arrest suggests that Hh-related signaling promotes transcription of genes associated with innate immunity. These innate immunity genes function downstream of Hh-related signaling to promote starvation-induced abnormalities. Furthermore, we report that extended L1 arrest leads to induction of innate immunity during late larval development without pathogen exposure, resulting in increased resistance to bacterial and intracellular pathogens. This work is significant for implicating additional genes and pathways in development of tumors following early life starvation. This work is also important for revealing an apparent tension between developmental robustness and anticipation of pathogen exposure during recovery from starvation, adding an additional dimension to the complex interplay between development and the environment.

## Results

### *daf-18/PTEN* suppresses starvation-induced gonad abnormalities independently of insulin/IGF signaling

**Fig 1:**
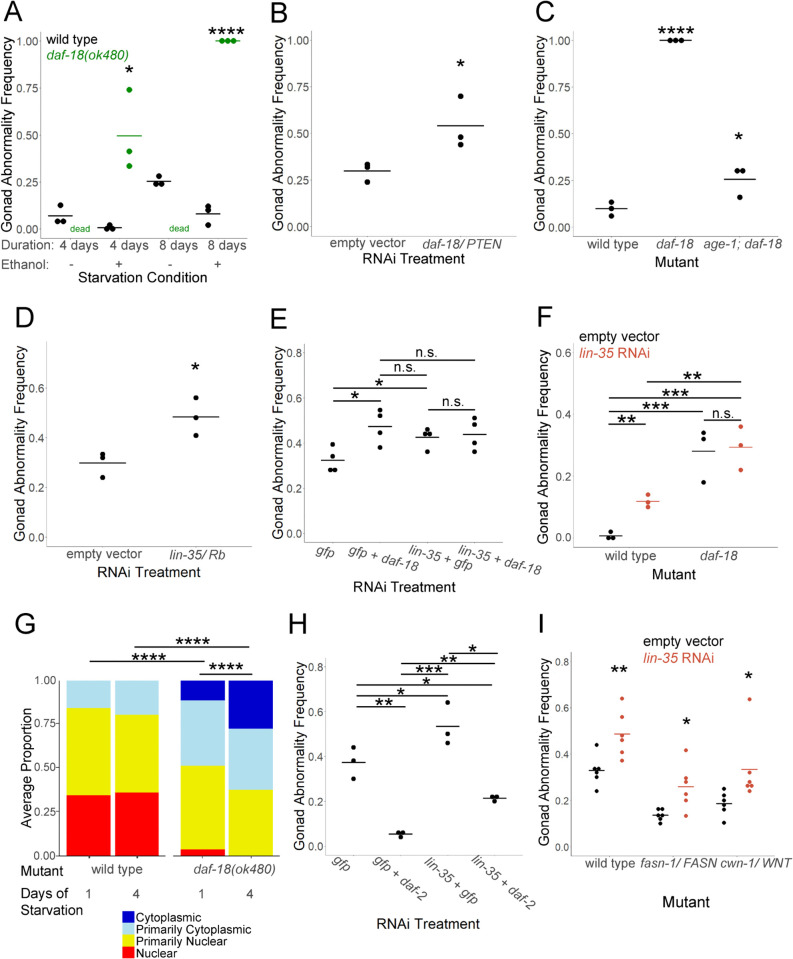
*daf-18/PTEN* and *lin-35/Rb* suppress starvation-induced gonad abnormalities independently of IIS. A-F, H, I) Gonad abnormalities (including proximal germ cell tumors, uterine masses, or other obvious developmental abnormalities in the gonad) were scored in various genotypes and conditions on the first day of egg laying. Starved worms were recovered with *E. coli* HT115 RNAi bacteria carrying an empty vector. Each dot represents a biological replicate of ~50 individuals (see S1 Table for summary statistics). Horizontal bars represent the mean across replicates. G) GFP::LIN-35 subcellular localization was scored for ~100 larvae in the indicated genotypes and L1 starvation conditions after 48 hours of recovery. Localization was scored as nuclear, primarily nuclear, primarily cytoplasmic, or cytoplasmic in intestinal cells in three biological replicates. Average frequency across replicates for each category is displayed. See also S1G. A) Wild type and *daf-18(ok480)* mutants were starved for 4 or 8 days during L1 arrest in S-basal with or without 0.1% ethanol. *daf-18(ok480)* does not survive 4 or 8 days of starvation without ethanol (“dead”). B, D, E, H, I) Gonad abnormalities were scored after 8 days of L1 arrest in virgin S-basal (no ethanol or cholesterol). B, D, E, H) Wild-type worms were starved and recovered on empty vector or the indicated RNAi bacteria. E, H) *gfp* RNAi was used as a negative control for double RNAi. C) Wild-type, *daf-18(ok480)*, and *age-1(m333); daf-18(ok480)* worms were starved in S-basal with 0.1% ethanol. *daf-18* vs. *age-1; daf-18* p-value = 9.57e-05. F) Wild-type and *daf-18(ok480)* worms were starved for four days in S-basal with 0.1% ethanol and recovered on empty vector or *lin-35* RNAi. I) Wild-type, *fasn-1(g14)*, and *cwn-1(ok546)* worms were recovered with empty vector or *lin-35* RNAi bacteria. A-I) Asterisks indicate statistical significance as determined with an unpaired t-test (A-F, H, I) or a Cochran-Mantel-Haenszel chi-squared test (G) compared to wild type within the same starvation condition (A), compared to empty vector (B, D), compared to wild type (C), as indicated by horizontal bars (E-H), and compared to empty vector (I). * P < 0.05, ** P < 0.01, **** P < 0.0001, “n.s.” = not significant.

*daf-18/PTEN* mutants are very sensitive to starvation and die rapidly during L1 arrest, but ethanol increases survival [18,22]. Wild-type larvae subjected to extended L1 arrest (*i.e*., 8 days) develop proximal germ cell tumors, differentiated uterine masses, and other gonad abnormalities [6–9] as illustrated in S1A and S1B Fig. With just 4 days of L1 starvation supplemented with ethanol, ~ 50% of *daf-18(ok480)* null mutant individuals developed one or more gonad abnormalities compared to almost none of the wild type (Fig 1A). After 8 days of starvation with ethanol, 100% of *daf-18* mutant worms developed abnormalities compared to ~10% of wild type. These results show that *daf-18* is a potent suppressor of starvation-induced gonad abnormalities, including germ cell tumors and uterine masses. However, given that *daf-18* supports starvation survival, it was unclear from these results whether *daf-18* functions during L1 arrest and/or recovery to suppress abnormalities. We starved wild-type larvae for 8 days without ethanol and recovered them with *daf-18* RNAi bacteria. The frequency of starvation-induced abnormalities was significantly increased (Fig 1B), showing that in addition to its role in starved L1 larvae, *daf-18* functions in fed, developing larvae to suppress starvation-induced abnormalities.

Disruption of *daf-2/InsR* suppresses starvation-induced gonad abnormalities (S1C Fig) [6–9], and we wondered if the potent effect of *daf-18/PTEN* on such abnormalities is due to its inhibition of IIS. We confirmed [6] that the transcription factors DAF-16/FoxO and SKN-1/Nrf, effectors of IIS, are required for *daf-2/InsR* RNAi to suppress starvation-induced abnormalities (S1C Fig). However, disruption of *daf-16* and *skn-1* alone or together did not significantly increase the frequency of abnormalities, though *daf-18* RNAi did (Fig 1B). Furthermore, a gain-of-function allele of *skn-1* and over-expression of *daf-16* incompletely suppressed abnormalities (S1D Fig). DAF-16 and the C2H2-type zinc finger transcription factor PQM-1 engage in mutual antagonism downstream of DAF-2 [23], and PQM-1 could function as an additional transcriptional effector of IIS. However, *pqm-1* RNAi did not significantly affect development of gonad abnormalities in previously starved worms (S1E Fig). Together these results suggest that changes in activity of the known effectors of IIS cannot account for the full effect of disruption of *daf-18/PTEN* on starvation-induced abnormalities.

PTEN dephosphorylates PIP3 to produce PIP2, inhibiting PI3K/IIS in mammals and *C. elegans*, but it can also dephosphorylate proteins [17,20]. PIP3 is not detectable in an *age-1/PI3K* null mutant [24], so if the only function of DAF-18 in suppressing starvation-induced abnormalities is to inhibit PI3K signaling via its lipid-phosphatase activity, then loss of *age-1/PI3K* should rescue the enhanced abnormality phenotype of *daf-18(ok480)* to wild-type penetrance or less. The *age-1(m333); daf-18(ok480)* double null mutant displayed a significantly lower gonad abnormality frequency than *daf-18(ok480)*, reflecting inhibition of PI3K/IIS via its lipid-phosphatase activity (Fig 1C). However, suppression was incomplete, and gonad abnormalities occurred at a significantly higher frequency in *age-1(m333); daf-18(ok480)* than wild type following starvation. This result suggests that in addition to inhibiting PI3K/IIS, DAF-18/PTEN suppresses starvation-induced gonad abnormalities independently of PI3K/IIS.

### *daf-18/PTEN* functions through *lin-35/Rb* to suppress starvation-induced gonad abnormalities

We recently discovered that *daf-18/PTEN* functions through *lin-35/Rb* to promote starvation survival during L1 arrest [21]. LIN-35 is the sole worm homolog of the retinoblastoma (RB) pocket protein family [25]. RB proteins are tumor suppressors [26], and we hypothesized that LIN-35/Rb suppresses formation of the proximal germ cell tumors and differentiated uterine masses which largely comprise the starvation-induced gonad abnormality phenotype. *lin-35* is required for vulva development [25]. Without L1 starvation, *lin-35(n745)* null mutant larvae develop gonad abnormalities and other defects, and they often do not survive to adulthood, so we did not analyze them. Instead, we recovered wild-type worms subjected to 8 days of L1 arrest with RNAi bacteria targeting *lin-35*, which significantly increased the frequency of starvation-induced abnormalities (Fig 1D). Notably, *daf-18* and *lin-35* RNAi did not cause gonad abnormalities without L1 starvation (S1F Fig), demonstrating that abnormalities depend on starvation and do not result from general disruption of development. These results show that *lin-35* functions in fed, developing larvae to suppress starvation-induced abnormalities, like *daf-18* (Fig 1B), suggesting *lin-35* could mediate the hypothetical PI3K/IIS-independent effect of *daf-18* on gonad abnormalities. Consistent with this hypothesis, disruption of *daf-18* and *lin-35* together did not increase the frequency of abnormalities compared to *lin-35* alone (Fig 1E). This RNAi-based result was corroborated with *lin-35* RNAi and the null mutant *daf-18(ok480)* (Fig 1F). In addition, *daf-18(ok480)* mutants displayed a shift in LIN-35 subcellular localization in the intestines of L4 larvae, with a significant increase in cytoplasmic LIN-35 compared to nuclear relative to wild type (Figs 1G and S1G). Since LIN-35 contributes to transcriptional regulation, this observation suggests *daf-18* promotes nuclear function of LIN-35, consistent with non-additive effects of their disruption (Fig 1E and 1F). Together these results suggest that DAF-18 acts through LIN-35 to suppress starvation-induced gonad abnormalities.

We analyzed genetic interactions between *lin-35/Rb* and *daf-2/InsR* to test the hypothesis that *lin-35* functions independently of IIS, like *daf-18/PTEN*. *daf-2/InsR* RNAi suppressed starvation-induced abnormalities (Fig 1H), as expected [6,8,9], and *lin-35* RNAi enhanced abnormalities (Fig 1H), also as expected (Fig 1D and 1E). However, disrupting *daf-2/InsR* and *lin-35/Rb* together suppressed abnormality frequency compared to *lin-35* alone (Fig 1H), with a comparable effect size of *daf-2* RNAi whether in combination with *lin-35* or *gfp* RNAi, suggesting independent effects of IIS and *lin-35/Rb* on development of starvation-induced abnormalities. In other words, disrupting *lin-35* and *daf-2* together enhanced abnormality frequency compared to *daf-2* alone (Fig 1H), with a comparable effect size of *lin-35* RNAi whether in combination with *daf-2* or *gfp* RNAi, again suggesting independent effects of IIS and *lin-35/Rb* on development of starvation-induced abnormalities.

The sole fatty acid synthetase *fasn-1/FAS* and WNT signaling function downstream of IIS to promote starvation-induced abnormalities [7,8]. *lin-35* RNAi significantly increased the frequency of abnormalities in *fasn-1/FAS* and *cwn-1/WNT* mutant backgrounds (Fig 1I), consistent with independent function of *lin-35*. These results further suggest that *lin-35/Rb* functions downstream of *daf-18/PTEN* and independently of PI3K/IIS to suppress starvation-induced gonad abnormalities.

### Hh-related signaling promotes starvation-induced gonad abnormalities downstream of *daf-18/PTEN* and *lin-35/Rb*

**Fig 2:**
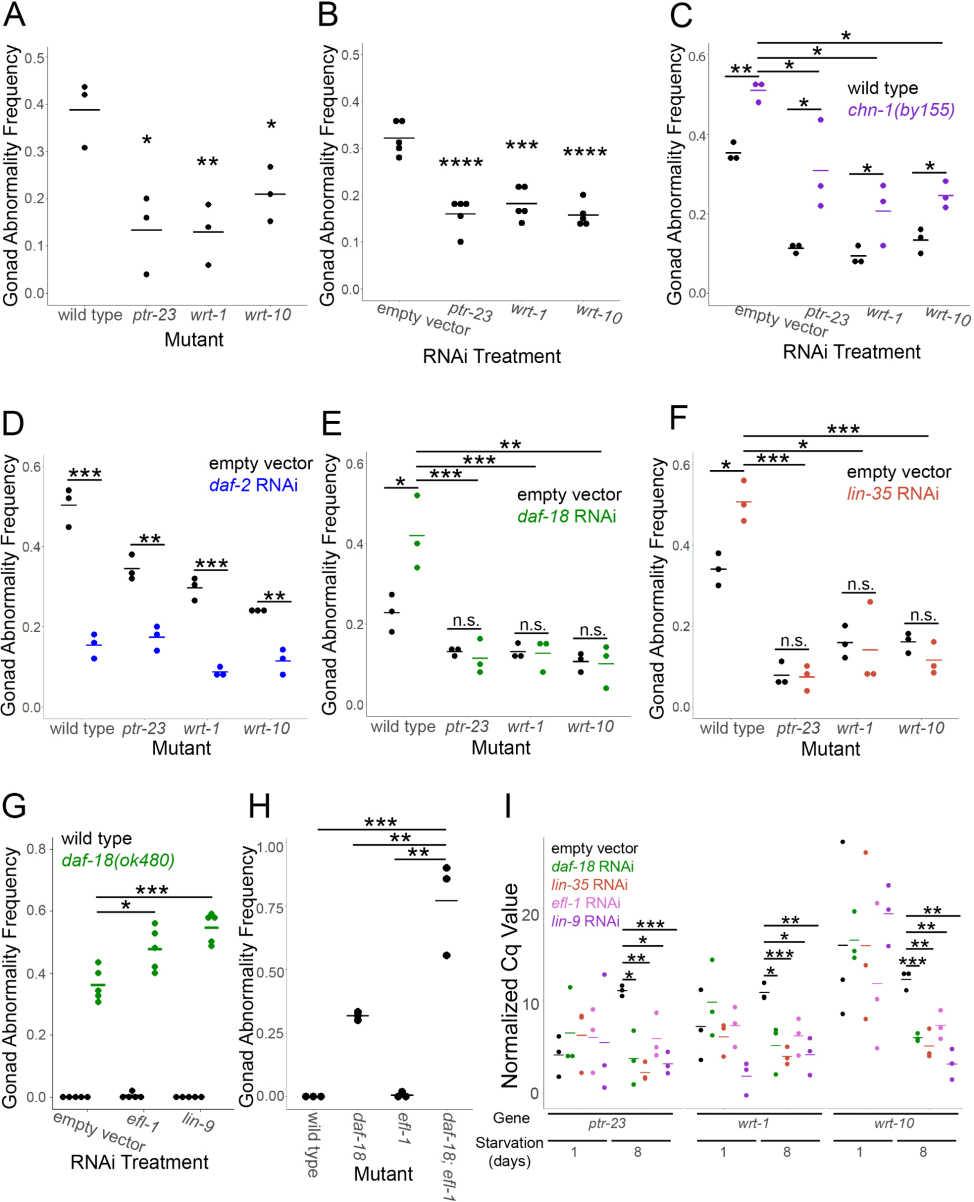
Hh-related genes promote starvation-induced gonad abnormalities downstream of *daf-18/PTEN* and *lin-35/Rb.* A-H) Larvae were starved in L1 arrest for 8 days in virgin S-basal (S-basal without ethanol or cholesterol) (A-F) or 4 days in S-basal with 0.1% ethanol (G, H), and gonad abnormalities were scored. Each dot represents a biological replicate with ~50 individuals (see S1 Table for summary statistics). I) RNA from wild-type larvae starved for 1 or 8 days in virgin S-basal recovered on the indicated RNAi strains was used for RT-qPCR and the mean Cq value for two technical replicates normalized to the housekeeping gene *tct-1* are plotted for the indicated genes. A) Wild type and *ptr-23(ok3663)*, *wrt-1(tm1417)*, and *wrt-10(aus36)*) were recovered on empty vector RNAi bacteria. B) Wild-type larvae were recovered on empty vector RNAi bacteria and RNAi targeting *ptr-23*, *wrt-1*, and *wrt-10*. C) Wild type and *chn-1(by155)* were recovered on empty vector RNAi bacteria and RNAi bacteria targeting *ptr-23*, *wrt-1*, and *wrt-10*. D-F) Wild type and *ptr-23*, *wrt-1*, and *wrt-10* mutants were recovered on empty vector RNAi bacteria and *daf-2* RNAi (D), *daf-18* RNAi (E), and *lin-35* RNAi (F). G) Wild type and *daf-18(ok480)* were recovered on empty vector RNAi bacteria and RNAi bacteria targeting *efl-1* or *lin-9*. A-I) Horizontal bars represent the mean across replicates. Asterisks indicate statistical significance relative to wild type (A), empty vector RNAi (B), and as indicated by bars (C-I). * P < 0.05, ** P < 0.01, *** P < 0.001, **** P < 0.0001, “n.s.” = not significant; unpaired, two-tailed t-test.

*daf-2/InsR*, *daf-16/FoxO*, *daf-18/PTEN*, *lin-35/Rb*, *fasn-1/FAS*, and WNT signaling all modulate formation of starvation-induced gonad abnormalities, and their homologs affect tumor development in mammals, suggesting that homologs of other genes that promote or suppress tumor formation could affect starvation-induced abnormalities [27–31]. Like WNT signaling, Hedgehog (Hh) signaling promotes stem cell proliferation and tumor formation [32–34]. We hypothesized that Hh signaling promotes starvation-induced gonad abnormalities. Most genes involved in Hh signaling have many homologs in *C. elegans*, some homologs are missing (*e.g.*, Smoothened), and in some cases there are missing or additional protein domains [35]. Consequently, it is relatively mysterious how Hh-related signaling works in *C. elegans*, though a variety of phenotypes related to molting, reproductive aging, germ cell proliferation, and innate immunity have been reported [36–40]. Expression analysis suggested that the PATCHED/PTCH receptor-related gene *ptr-23* and the Hedgehog-related genes *wrt-1* and *wrt-10* are upregulated in adults by early life starvation [7]. We found that mutation of each of these genes suppressed starvation-induced gonad abnormalities (Fig 2A). *ptr-23*, *wrt-1*, and *wrt-10* mutants did not survive longer than wild type during L1 arrest (S2A Fig), suggesting the decrease in abnormality frequency is not due to a general increase in starvation resistance in the mutants. RNAi of these genes during recovery from L1 arrest also suppressed starvation-induced abnormalities (Fig 2B), suggesting that Hh-related signaling during larval development promotes formation of starvation-induced gonad abnormalities.

Having established a role for Hh-related signaling in development of starvation-induced gonad abnormalities, we asked whether Hh-related signaling functions independently of IIS. The ubiquitin ligase CHN-1/CHIP ubiquitinates DAF-2/InsR promoting its degradation, and loss of *chn-1/CHIP* increases DAF-2 abundance and IIS [41]. The *chn-1(by155)* null mutant increased the frequency of starvation-induced abnormalities (Fig 2C), as expected [9]. The mutant also increased abnormality frequency along with RNAi targeting *ptr-23*, *wrt-1*, and *wrt-10*, suggesting that Hh-related signaling functions independently of IIS. *chn-1* mutant larvae did not develop gonad abnormalities without extended L1 arrest (S2B Fig), demonstrating that abnormalities depend on starvation and do not result from general disruption of development. CHN-1 may have targets in addition to DAF-2. However, *daf-2* RNAi suppressed abnormality frequency in *ptr-23*, *wrt-1*, and *wrt-10* mutants (Fig 2D), supporting the conclusion that Hh-related signaling functions independently of IIS to promote starvation-induced gonad abnormalities.

Given that Hh-related signaling functions independently of IIS in this context, we hypothesized that it functions downstream of *daf-18/PTEN* and *lin-35/Rb*. *daf-18* RNAi and *lin-35* RNAi did not increase penetrance of starvation-induced abnormalities in *ptr-23*, *wrt-1*, or *wrt-10* mutant backgrounds (Fig 2E and 2F), suggesting that these Hh-related signaling genes are epistatic to *daf-18* and *lin-35*. These results support the conclusion that Hh-related signaling promotes starvation-induced gonad abnormalities downstream of *daf-18/PTEN* and *lin-35/Rb*.

### The DREAM complex represses transcription of Hh-related genes to suppress starvation-induced gonad abnormalities

RB family proteins repress cell cycle-dependent transcription in quiescent mammalian cells [42]. RB proteins bind to E2F and DP family members [43]. The protein encoded by *RB1*, pRB, represses transcription together with E2F/DP [44]. In addition, the protein products of *RBL1* and *RBL2* (p107 and p130, respectively) recruit MuvB proteins [45], and RB, E2F, and DP family proteins plus five MuvB proteins form the DREAM complex, which also mediates transcriptional repression [42]. Functions of these proteins is conserved in *C. elegans*, with LIN-35/Rb mediating association between E2F/DP and MuvB proteins, stabilizing the DREAM complex [46], which binds chromatin to repress transcription [47,48]. In addition, LIN-35 and the rest of DREAM promote starvation survival during L1 arrest [21].

We hypothesized LIN-35/Rb acts with E2F and possibly the rest of DREAM to suppress starvation-induced abnormalities. We assayed *efl-1*, which encodes one of the *C. elegans* homologs of mammalian E2F, and *lin-9*, which encodes one of the five *C. elegans* MuvB proteins. In contrast to *lin-35* (Fig 1D), RNAi of *efl-1* and *lin-9* did not increase the frequency of starvation-induced abnormalities (Fig 2G). However, RNAi of *efl-1* and *lin-9* significantly increased abnormality frequency in *daf-18(ok480)* (Fig 2G), revealing non-additivity between *daf-18/PTEN* and *lin-9.* Furthermore, *daf-18; efl-1* double mutants displayed robust non-additivity (Fig 2H), corroborating RNAi. In this case, non-additivity is due to ‘synergistic’ epistasis, as opposed to ‘antagonistic’. That is, disruption of both genes produces a more penetrant phenotype than the sum of the two individual perturbations. These results suggest that *daf-18* and the DREAM complex function in a common pathway, with loss of *daf-18* incompletely disrupting DREAM function and *daf-18* suppressing starvation-induced abnormalities through an independent mechanism as well (*i.e.*, inhibition of IIS).

We hypothesized that LIN-35/Rb and DREAM repress transcription of Hh-related genes to suppress starvation-induced abnormalities. We recovered wild-type L1 larvae after 1 or 8 days of L1 arrest on RNAi bacteria targeting *daf-18, lin-35, efl-1,* or *lin-9* and performed RT-qPCR in L4 larvae (48 hr recovery). None of the perturbations had a significant effect on expression of *ptr-23, wrt-1,* or *wrt-10* following 1 day of L1 arrest (Fig 2I). However, all four perturbations resulted in significantly lower Cq values and therefore increased transcript abundance for all three genes following 8 days of L1 arrest (Fig 2I). These results suggest that LIN-35/Rb and DREAM repress transcription of Hh-related genes following extended L1 starvation, and they further support the conclusion that DAF-18/PTEN acts through LIN-35 and DREAM to suppress starvation-induced abnormalities.

### Hh-related genes function in the epidermis and intestine to promote starvation-induced gonad abnormalities

**Fig 3:**
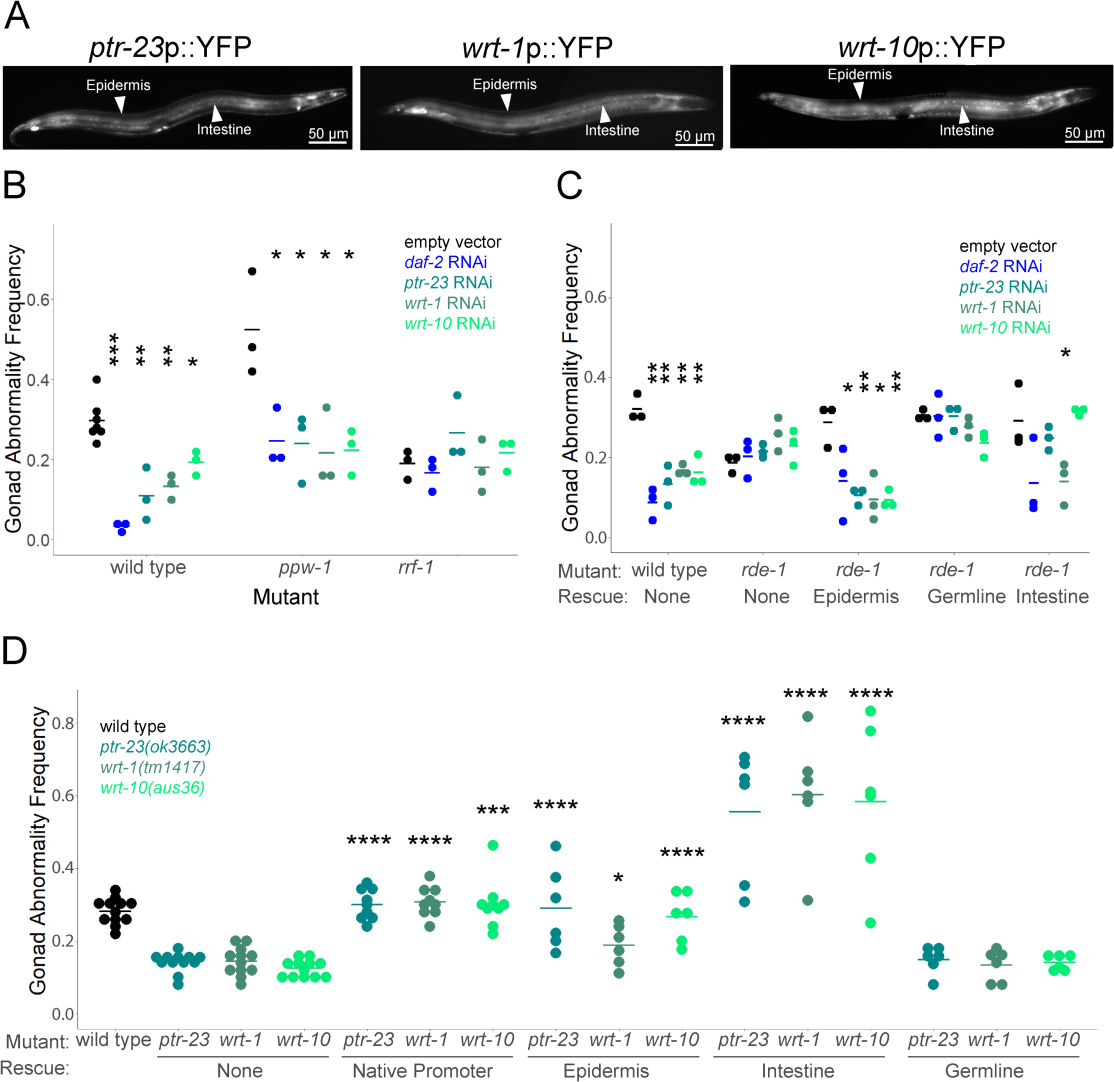
Hh-related genes function in the epidermis and intestine to promote starvation-induced gonad abnormalities. A) Representative images of *ptr-23*p::YFP, *wrt-1*p::YFP, and *wrt-10*p::YFP at 400X total magnification show expression primarily in the epidermis and intestine as indicated with arrowheads and labels. Images were taken in L4 larvae (48 hours after hatching) in well-fed individuals. B) Wild type, *ppw-1(pk2505),* and *rrf-1(pk1417)* L1 larvae were arrested for 8 days as L1 larvae then recovered on empty vector, *daf-2*, *ptr-23, wrt-1,* or *wrt-10* RNAi bacteria and scored for gonad abnormalities. C) Wild type and *rde-1(ne219)* mutants were starved for 8 days then recovered on empty vector, *ptr-23, wrt-1,* and *wrt-10* RNAi and scored for gonad abnormalities. *rde-1(ne219)* null mutants that had been rescued with a multicopy *rde-1* transgene with tissue-specific expression in the epidermis (*lin-26p*), germline (*mex-5p*), or intestine (*nhx-2*) were also included. D) Wild type and *ptr-23(ok3663), wrt-1(tm1417),* and *wrt-10(au36)* were starved for 8 days as L1 larvae then recovered on empty vector RNAi bacteria and scored for gonad abnormalities. *ptr-23(ok3663), wrt-1(tm1417),* and *wrt-10(au36)* mutants that had been rescued with a multicopy transgene with their native promoter or a tissue-specific promoter for the epidermis (*col-12p*), intestine (*ges-1p*), or germline (*mex-5p*), were also scored. Gonad abnormalities are significantly greater than wild type with intestinal rescue of each Hh-related gene (P < 0.0001). B-D) Each dot represents a biological replicate with ~50 individuals (see S1 Table for summary statistics). Horizontal bars represent the mean across replicates. Asterisks indicate significance between the indicated RNAi treatment and empty vector RNAi bacteria within the same genotype (B-C), and between the indicated mutants with transgenic rescue and the corresponding mutant without rescue (“none”) (D). * P < 0.05, ** P < 0.01, *** P < 0.001, **** P < 0.0001; Unpaired, two-tailed t-test.

We made translational reporter genes for *ptr-23*, *wrt-1*, and *wrt-10* to determine where they are expressed during larval development. Each reporter gene was expressed throughout the epidermis (Fig 3A), consistent with single-cell mRNA sequencing (scRNA-seq) of L2 larvae [49] and reporter gene analysis of *ptr-23* [50]. Each reporter gene was also expressed throughout the intestine, though only *ptr-23* had substantial expression there according to scRNA-seq [49]. Dim expression was also observed in the pharynx for *ptr-23*p::YFP and *wrt-10*p::YFP, and in the vulva for *ptr-23*p::YFP, consistent with scRNA-seq [49]. Additional unidentified cells in the head and tail were also observed.

We used multiple complementary approaches to determine the anatomical site(s) of action for *ptr-23*, *wrt-1*, and *wrt-10* in promoting starvation-induced gonad abnormalities. *ppw-1* and *rrf-1* mutants are generally deficient for germline and somatic RNAi, respectively [51,52]. *daf-2/InsR* RNAi suppressed abnormalities in wild-type and *ppw-1* mutants but not *rrf-1* (Fig 3B), as expected [6]. RNAi is partially effective in some somatic tissues in *rrf-1* mutants [53], but this result nonetheless suggests that *daf-2* functions in the soma, as opposed to the germline, to suppress abnormalities, as shown elsewhere [6]. Likewise, RNAi of *ptr-23*, *wrt-1*, and *wrt-10* suppressed abnormalities in wild-type and *ppw-1* mutant backgrounds but not the *rrf-1* background (Fig 3B), suggesting that Hh-related signaling functions in the soma to promote gonad abnormalities.

*rde-1* is required for RNAi throughout the animal [54], and we used strains with tissue-specific transgenic rescue of an *rde-1* mutation to further evaluate potential site(s) of action of *ptr-23*, *wrt-1*, and *wrt-10.* Mutation of *rde-1* abrogated the effects of RNAi for *daf-2* and each of the Hh-related genes (Fig 3C), as expected. Rescue of *rde-1* in the epidermis (*lin-26p*) [55] restored suppression of starvation induced-abnormalities following *ptr-23*, *wrt-1*, and *wrt-10* RNAi (Fig 3C), consistent with prominent expression of all three genes in the epidermis [49]. Intestinal rescue (*nhx-2p*) [56] produced mixed results, with *wrt-1* RNAi suppressing abnormalities but not *ptr-23* or *wrt-10.* Germline rescue (*mex-5p*) [57] of *rde-1* did not result in suppression of abnormalities with RNAi of any gene tested (Fig 3C), consistent with analysis of *ppw-1* and *rrf-1* mutants (Fig 3B). These results suggest that all three Hh-related genes function in the epidermis and that *wrt-1* also functions in the intestine to suppress abnormalities.

Transgenic rescue of *ptr-23*, *wrt-1*, and *wrt-10* with their own promoters restored wild-type penetrance of gonad abnormalities (Fig 3D), as expected. Epidermal rescue (*col-12p*) of each gene also increased the penetrance of starvation-induced abnormalities, but germline rescue (*mex-5p*) did not, consistent with tissue-specific RNAi (Fig 3B and 3C). Surprisingly, intestinal rescue (*ges-1p*) of all three genes increased penetrance above wild-type levels (Fig 3D), likely due to over-expression with a heterologous promoter and multi-copy transgene. Intestinal rescue did not cause gonad abnormalities without extended L1 arrest (S3 Fig), demonstrating that abnormalities depend on starvation and do not result from general disruption of development. Together with our tissue-specific RNAi results, these results suggest that *wrt-1* is necessary and sufficient in both epidermis and intestine to promote starvation-induced gonad abnormalities, and that *ptr-23* and *wrt-10* are necessary and sufficient in the epidermis but merely sufficient in the intestine to do so. Moreover, these results suggest cell-nonautonomous effects of Hh-related signaling on germ cell proliferation and other aspects of gonad development affected by extended L1 arrest.

### Hh-related genes promote expression of genes related to innate immunity

**Fig 4:**
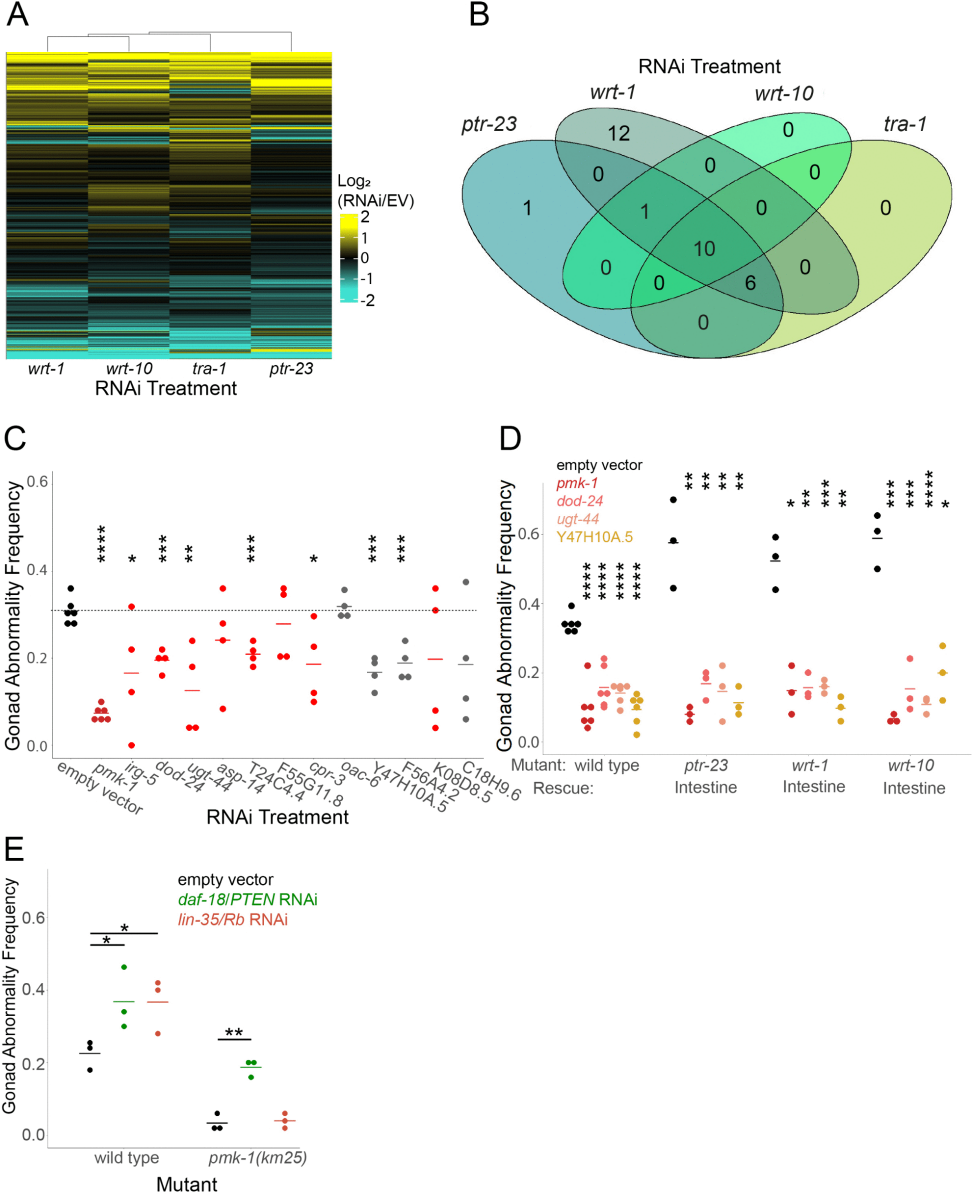
Hh-related signaling promotes innate immunity gene expression to promote starvation-induced gonad abnormalities. A) RNA-seq was performed on the first day of egg laying in worms that had been subjected to 8 days of starvation during L1 arrest and then recovered with empty vector, *ptr-23, tra-1, wrt-1,* or *wrt-10* RNAi bacteria. Log_2_ fold-changes of transcript abundance between RNAi of each gene and empty vector were clustered and are presented as a heat map. Each horizontal bar represents expression of a gene. A generalized linear model was used to identify differentially expressed genes across all four treatments (FDR < 0.05), and these genes were used for cluster analysis (n = 365). See S3 Table for RNA-seq results. B) The number of genes that were differentially expressed in each pairwise comparison between RNAi and empty vector is plotted in a Venn diagram. An exact test (EdgeR) was used to identify differentially expressed genes (FDR < 0.1). C) Wild type L1 larvae were starved for 8 days then recovered with empty vector RNAi bacteria, *pmk-1* RNAi (positive control), or RNAi against twelve of the down-regulated genes from Table 1, then scored for gonad abnormalities on the first day of egg laying. Genes identified by WormCat as components of the innate immune system are in red. The four genes in gray were upregulated by PA14 exposure and downregulated by loss of *pmk-1/p38 MAPK* [60]. D) Wild type and *ptr-23(ok3663), wrt-1(tm1417),* and *wrt-10(au36)* with intestinal rescue (Fig 3D) were starved for 8 days as L1 larvae and recovered with empty vector RNAi bacteria, *pmk-1* RNAi, or RNAi against three of the genes analyzed in C. E) Wild type and *pmk-1(km25)* were starved for 8 days as L1 larvae and recovered with the indicated RNAi bacteria. C-E) Each dot represents a biological replicate with ~50 individuals (see S1 Table for summary statistics). Horizontal bars represent the mean across replicates. Asterisks indicate significance between the indicated RNAi treatment and empty vector RNAi (C) or empty vector RNAi within the same genotype (D, E). * P < 0.05, ** P < 0.01, *** P < 0.001, **** P < 0.0001; unpaired, two-tailed t-test.

We used RNA sequencing (RNA-seq) of whole adult worms to identify genes whose expression is affected by Hh-related signaling following extended L1 arrest. We starved wild-type L1 larvae for 8 days, recovered them with empty vector RNAi bacteria or RNAi targeting *ptr-23*, *wrt-1*, *wrt-10*, or *tra-1/GLI*, collected them on the first day of egg laying, and prepared RNA for sequencing. *tra-1* encodes the sole worm homolog of the GLI transcription factor family [58]. GLI factors function as transcriptional effectors of canonical Hh signaling [59]. Although *tra-1/GLI* has not been shown to function in Hh-related signaling, we included it in our RNA-seq experiment since it was unclear how the other Hh-related genes could affect transcription. Notably, *tra-1* RNAi suppressed starvation-induced abnormalities (S4 Fig), suggesting it could mediate effects of *ptr-23*, *wrt-1*, and *wrt-10.* Cluster analysis of 365 genes differentially expressed across the entire set of conditions (generalized linear model) suggested that all four Hh-related genes had similar effects on gene expression (Fig 4A). Relatively few genes were significantly differentially expressed in any one perturbation relative to the control (exact test), but a majority of the differentially expressed genes were affected by three or four of the perturbations (Fig 4B), and in the same direction. Together these results suggest that the Hh-related genes converge on transcriptional regulation of a common set of genes.

**Table 1:** Differentially expressed genes due to disruption of Hh-related signaling.

ORF	Log₂FC (RNAi/EV)	FDR	WormCat	Activation by PA14	Activation by *pmk-1*
F35E12.5	−2.1	5.5E-10	Stress response: pathogen	Yes	Yes
F21F8.4	−2.0	1.1E-07	NA	No	No
Y39B6A.24	−1.9	7.2E-16	NA	No	No
C32H11.12	−1.9	1.9E-07	Stress response: pathogen	Yes	Yes
F01D4.2	−1.4	0.005	NA	Yes	Yes
F56D6.2	−1.3	3.2E-08	Stress response: C-type Lectin	No	
K10C2.3	−1.3	3.7E-07	NA	Yes	No
T24C4.4	−1.3	0.06	Stress response: pathogen	Yes	Yes
C17H12.8	−1.2	1.09E-13	NA	No	No
F55G11.8	−1.2	0.001	Stress response: pathogen	Yes	Yes
F54B11.11	−1.1	0.08	Stress response: pathogen	No	No
T10H4.12	−1.0	0.01	Stress response: pathogen	Yes	Yes
C31A11.5	−1.0	0.01	NA	Yes	Yes
Y47H10A.5	−0.9	0.005	NA	No	No
F55G11.4	−0.9	0.001	Stress response: pathogen	Yes	Yes
Y19D10A.9	−0.9	1.8E-13	Stress response: C-type Lectin	Yes	Yes
T19D12.4	−0.8	0.0009	Stress response: pathogen	No	No
F56A4.2	−0.8	1.2E-09	Stress response: C-type Lectin	Yes	Yes
K08D8.4	−0.8	0.09	Stress response: pathogen	No	No
K08D8.5	−0.7	0.03	Stress response: pathogen	Yes	Yes
C05A9.1	−0.7	0.03	NA	No	No
Y54G11A.5	−0.7	1.7E-06	NA	No	No
Y54G2A.6	−0.6	0.001	Stress response: C-type Lectin	No	No
C02A12.4	−0.6	0.03	Stress response: pathogen	No	No
C18H9.6	−0.6	0.0006	NA	Yes	Yes
F15E11.14	−0.6	0.05	NA	Yes	Yes
R06B10.3	−0.6	3.8E-06	Stress response: C-type Lectin	No	No
F49E12.2	−0.6	2.8E-05	NA	No	No
F27C8.4	−0.5	0.09	Stress response: pathogen	No	No
F35E12.6	−0.5	0.05	Stress response: pathogen	No	No
ZK6.10	−0.5	0.03	Stress response: pathogen	No	No
C14A6.1	−0.5	0.01	Stress response: C-type Lectin	No	No
W04E12.8	−0.4	3.1E-06	Stress response: C-type Lectin	No	No
Y119D3B.21	−0.4	0.05	NA	No	No
ZK6.11	−0.4	0.005	Stress response: pathogen	No	No
F35C5.6	−0.4	0.01	Stress response: C-type Lectin	No	No
Y54G11A.6	−0.4	0.03	NA	No	No
F32A5.5	−0.4	0.07	NA	No	No
T21H3.1	−0.3	0.01	NA	No	No
C54G6.5	−0.3	0.01	NA	No	No
D2096.3	−0.3	0.06	NA	No	No
F44C4.3	0.3	0.04	NA	No	No
Y54G11A.7	0.3	0.03	NA	No	No
W07G4.5	0.4	0.08	NA	No	No
Y40D12A.2	0.4	0.03	NA	No	No
F42G2.2	0.4	0.09	NA	No	No
K10B2.2	0.5	0.002	NA	No	No
C33A12.6	0.6	0.04	NA	No	No
F59D6.3	0.6	0.0005	NA	No	No
C29E4.7	0.8	3.01E-10	NA	No	No
Y51A2D.4	0.9	0.04	NA	No	No
F36A2.3	1.0	1.9E-08	NA	No	No
Y50E8A.16	1.0	9.4E-11	NA	No	No
C40H1.2	1.1	0.1	NA	No	No
K01D12.14	1.2	1.9E-08	NA	No	No
C40H1.8	1.3	0.002	NA	No	No
F58E6.8	1.4	0.05	NA	No	No
F36A4.5	1.4	0.009	NA	No	No
C47A10.12	1.8	0.04	NA	No	No
F18A12.4	3.4	1.7E-26	NA	No	No

All replicates for all RNAi treatments (*wrt-1*, *wrt-10*, *ptr-23*, and *tra-1*) were combined and treated as a single perturbation to compare to empty vector to identify a larger set of differentially expressed genes with the exact test. The resulting list of differentially expressed genes (FDR < 0.1) was sorted by fold change and is shown. Enrichment of WormCat categories associated with innate immunity and C-type lectins are indicated. Genes transcriptionally activated by *P. aeruginosa* PA14 exposure and *pmk-1* as identified in [60] are also indicated.

We treated all replicates for all four RNAi treatments as a single perturbation to identify additional genes affected by Hh-related signaling. Strikingly, 22 of the 41 genes downregulated by Hh-related RNAi are associated with innate immunity when queried with the gene set enrichment tool WormCat [61] (Table 1). Fourteen downregulated genes are categorized as ‘Stress response: pathogen’ (p-value = 1.65E-18) and eight are categorized as ‘Stress response: C-type Lectin’ (p-value = 9.96E-08), which are associated with innate immunity [61,62], but none of the 19 upregulated genes are associated with innate immunity (Table 1). PMK-1/p38 MAPK is an essential component of a pathway that activates immunity in response to pathogens [63]. In another published study [60], *Pseudomonas aeruginosa* exposure activated 15 of the 41 downregulated genes, and all but one of those was activated by *pmk-1*, but none of the 19 upregulated genes were affected by either (Table 1). These gene expression results suggest that Hh-related signaling promotes expression of genes related to innate immunity.

### Innate immunity genes promote starvation-induced gonad abnormalities downstream of Hh-related signaling

We hypothesized that Hh-related signaling promotes expression of genes, specifically innate immunity genes, that promote starvation-induced gonad abnormalities. We targeted *pmk-1* with RNAi along with twelve genes downregulated by Hh-related RNAi, including eight genes associated with innate immunity according to WormCat and four that were not but were activated by *pmk-*1 and *P. aeruginosa* exposure. RNAi of *pmk-1* and seven of the other twelve genes tested significantly decreased the frequency of starvation-induced abnormalities (Fig 4C). These results suggest that genes associated with innate immunity, including its critical regulator *pmk-1/p38 MAPK*, promotes starvation-induced gonad abnormalities.

We took advantage of the elevated frequency of starvation-induced abnormalities resulting from intestinal overexpression of *ptr-23*, *wrt-1*, and *wrt-10* (Fig 3D) to determine if these immunity-related genes function downstream of Hh-related signaling. RNAi of *pmk-1* and the three other genes tested suppressed gonad abnormalities despite intestinal overexpression of *ptr-23*, *wrt-1*, and *wrt-10* (Fig 4D). Likewise, epistasis was observed between *pmk-1(km25)* and *lin-35* RNAi (Fig 4E). In contrast, *daf-18* and *pmk-1* had additive effects on starvation-induced abnormalities, which we attribute to DAF-18/PTEN inhibition of IIS. Together these results suggest that the *pmk-1/p38 MAPK* innate immunity pathway promotes starvation-induced gonad abnormalities downstream of *lin-35/Rb* and Hh-related signaling.

### Starvation during L1 arrest induces innate immunity and pathogen resistance later in life

**Fig 5:**
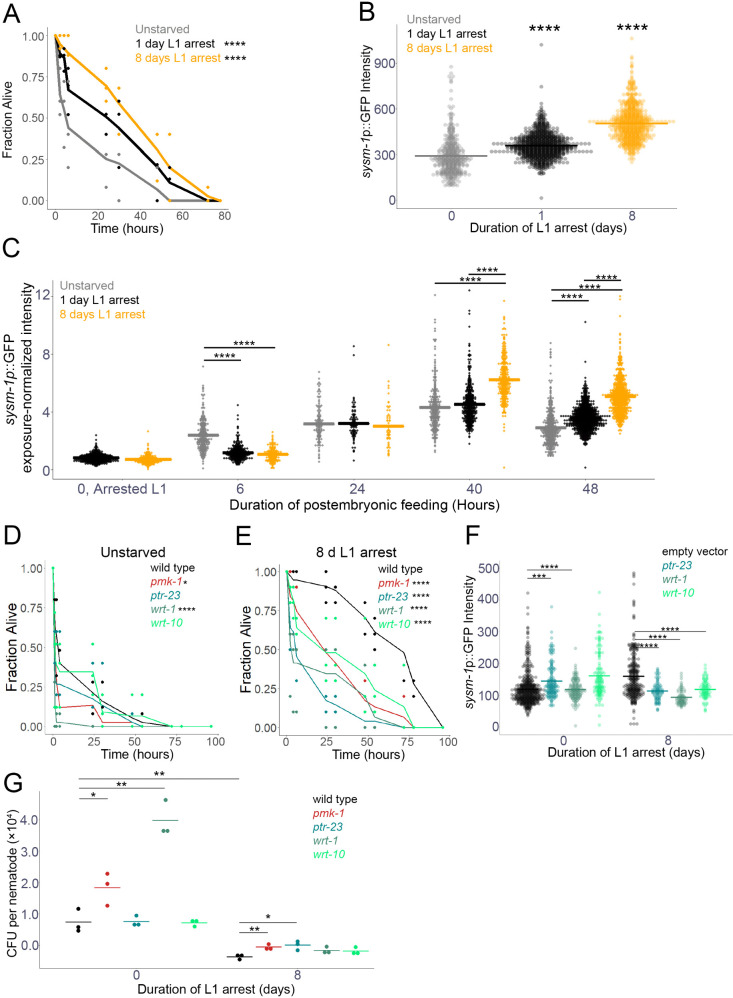
Starvation during L1 arrest promotes innate immunity later in life via Hh-related signaling. A) Wild-type L1 larvae were starved for the indicated duration, recovered on plates with *E. coli* HT115 bacteria, L4 larvae were transferred to *P. aeruginosa* PA14 “fast-killing” plates, and survival was assayed. See S4 Table for pathogen resistance summary statistics. B-C) L1 larvae with a *sysm-1p::gfp* reporter gene were starved for the indicated duration, recovered on plates with empty vector RNAi bacteria for 48 hr (B), or the indicated duration (C) and whole-worm reporter gene expression was quantified by image analysis. C) Due to varying exposure times, exposure-normalized whole worm GFP intensity (fluorescence intensity divided by exposure time) is reported. D, E) Unstarved worms (D) or worms that had been starved for 8 days as L1 larvae (E) were exposed to PA14 as in A, except that in addition to wild type, *pmk-1(km25)*, *ptr-23(ok3663)*, *wrt-1(tm1417)*, and *wrt-10(aus36)* were included. F) L1 larvae with a *sysm-1p::gfp* reporter gene were starved for 0 (unstarved) or 8 days, recovered, and imaged as in B, except that they were recovered with empty vector RNAi bacteria or RNAi targeting *ptr-23*, *wrt-1*, or *wrt-10*. G) Unstarved worms and worms that had been starved for 8 days as L1 larvae were exposed to GFP-labelled PA14 for two hours then lysed to extract bacteria colonized in the intestine. Lysates were spread on LB plates and incubated at 37^o^C overnight, then fluorescent colonies were counted to calculate the number of colony-forming units per individual worm. Individual points represent the average CFU count per biological replicate, each with 10 individuals. B, F) To ensure matching stages, unstarved control worms (0 hr) were allowed 8 additional hours to account for embryogenesis, and worms starved for 8 days were also allowed 8 additional hours to account for developmental delay following extended starvation. A, D, E) Three biological replicates were performed with ~50 individuals each. Mean survival at each timepoint is plotted as a line with survival of each replicate at each timepoint included as dots. Asterisks indicate significance between the indicated starvation durations (1 or 8 d L1 arrest) and unstarved controls (A) or between the indicated genotype and wild type (D, E). The log-rank test was used for pairwise comparisons. B, C, F) Individual points represent the average, background-corrected pixel intensity for a single worm. Horizontal bars represent the mean intensity across three biological replicates. A linear mixed-effect model was fit to the data with background-corrected average intensity as the response variable, RNAi treatment as the fixed effect, and replicate as the random effect. Asterisks and bars indicate significance. A-G) * P < 0.05, *** P < 0.001, **** P < 0.0001.

We were surprised to find that genes associated with innate immunity promote starvation-induced abnormalities, and we wondered if they function in the innate immunity pathway to do so. We reasoned that if they do, then starvation during L1 arrest should result in increased resistance to bacterial pathogens. Remarkably, L4 larvae starved for 1 or 8 days during L1 arrest displayed increased resistance to *P. aeruginosa* strain PA14 in a “fast-killing” assay [64] compared to control worms that had not been starved during L1 arrest (“unstarved”), with 8 days of starvation having a larger effect than 1 day (Fig 5A). Moreover, L4 larvae starved for 8 days during L1 arrest displayed increased resistance to PA14 in a “slow-killing” assay [64] and to *Salmonella enterica* (S5A and S5B Fig). We wondered if immunity extended beyond bacterial pathogens, and we found that 8 days of L1 arrest reduced infection upon exposure to microsporidia (S5C Fig), a eukaryotic intracellular pathogen [65].

*sysm-1* induction is a marker of the innate immune response [66]. 1 or 8 days of L1 arrest was sufficient to increase expression of a *sysm-1p::gfp* reporter gene in L4 larvae without pathogen exposure, with 8 days of starvation having a larger effect than 1 day (Figs 5B and S5D). To determine when the *sysm-1p::gfp* reporter is induced (*e.g.*, during starvation or recovery), we measured *sysm-1*p::GFP intensity in arrested L1 larvae and at different timepoints during recovery (Fig 5C). There was no difference in intensity in L1 larvae that had been starved for 1 or 8 days, suggesting that reporter gene induction does not occur as an immediate response to extended starvation. Expression of *sysm-1*p::GFP was significantly lower in previously starved larvae than unstarved controls at 6 hr, and it was equivalent between them at 24 hr. However, *sysm-1*p::GFP expression was significantly higher at 40 hr for larvae that had been starved for 8 days compared to unstarved controls, and it was significantly higher for larvae that had been starved for 1 or 8 days at 48 hr (Fig 5C), as expected (Fig 5B). These results suggest that exposure to food (non-pathogenic *E. coli*) during recovery from L1 arrest, provokes expression of *sysm-1*p::GFP, with a longer duration of starvation having a stronger effect, and that a substantial amount of recovery time is required for induction of immunity.

### Hh-related signaling is required for L1 arrest to result in bacterial pathogen resistance

Since Hh-related signaling promotes starvation-induced gonad abnormalities (Fig 2), innate immunity genes promote abnormalities downstream of Hh-related signaling (Fig 4), and L1 starvation induces innate immunity (Figs 5A,5B and S5A-C), we hypothesized that Hh-related signaling promotes innate immunity in response to L1 starvation. Mutations disrupting function of *pmk-1/p38 MAPK* and *wrt-1*, but not *ptr-23* or *wrt-10*, significantly decreased resistance to PA14 in L4 larvae that had not been subjected to L1 starvation (Fig 5D). In contrast, mutations affecting all four genes dramatically decreased resistance to PA14 in larvae that had been starved for 8 days (Fig 5E). Critically, the increase in resistance caused by extended L1 arrest was largely dependent on the Hh-related genes, with those mutants displaying pathogen sensitivity similar to *pmk-1*. Furthermore, RNAi of *ptr-23*, *wrt-1*, and *wrt-10* prevented upregulation of *sysm-1*p::GFP following 8 days of L1 arrest (Fig 5F). Together these results suggest that induction of resistance to *P. aeruginosa* in L4 larvae and adults by L1 arrest depends on Hh-related signaling. However, induction of resistance to microsporidia infection by extended L1 arrest did not depend on Hh-related signaling (S5C Fig), suggesting that early life starvation can alter pathogen susceptibility through alternative mechanisms.

Differences in survival during *P. aeruginosa* exposure could result from a variety of factors. The PA14 bacterial lawn was spread all over the plates for our survival assays, ruling out differences in pathogen avoidance contributing to differences in survival. Avoidance aside, differences in survival could be due to differences in pathogen tolerance or resistance. We measured PA14 colony forming units (CFUs) in exposed worms to quantify infection levels. The effects of extended starvation and *ptr-23*, *wrt-1*, and *wrt-10* mutants on infection levels echoed their effects on survival (Fig 5D and 5E), with extended starvation reducing infection and *pmk-1* and *wrt-1* mutants clearly increasing infection (Fig 5G). These results suggest that survival is affected by differences in pathogen resistance, though differences in tolerance could also contribute. Extended starvation and *ptr-23*, *wrt-1*, and *wrt-10* mutants did not have significant effects on pharyngeal pumping or defecation rates on *E. coli* or *P. aeruginosa* (S5E and S5F Fig), suggesting that differences in pathogen resistance are not due to effects on feeding or defecation behavior.

## Discussion

**Fig 6:**
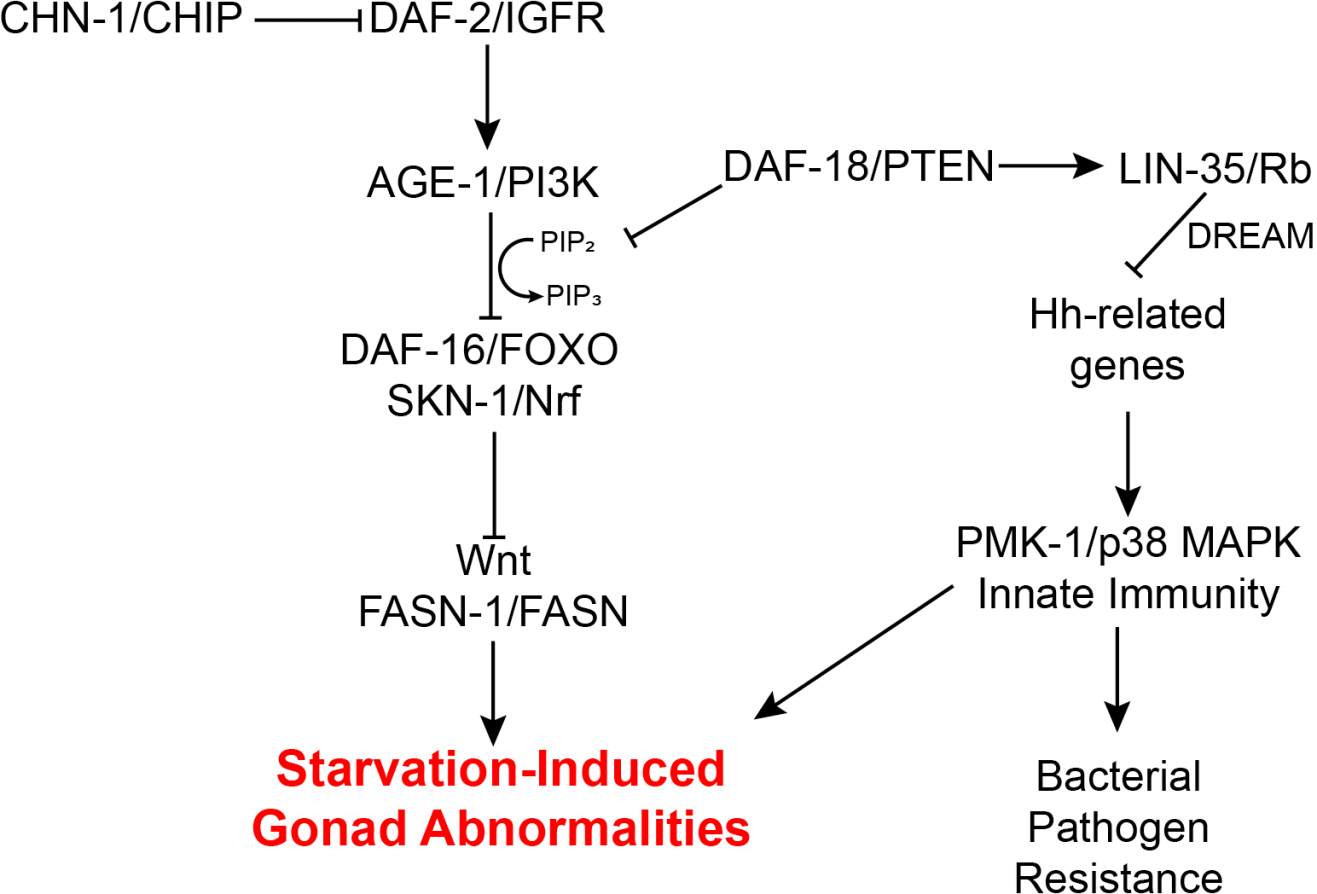
A model for how *daf-18/PTEN* and *lin-35/Rb* protect worms from developing L1 starvation-induced gonad abnormalities. Previous work has shown that reducing IIS suppresses starvation-induced abnormalities via *daf-16/FoxO* and *skn-1/Nrf*. We show that in addition to antagonizing IIS, *daf-18/PTEN* suppresses abnormalities via *lin-35/Rb*, which recruits the DREAM complex to repress transcription of Hh-related genes *wrt-1*, *wrt-10*, and *ptr-23*. We also show that Hh-related signaling promotes abnormalities by activating *pmk-1/p38 MAPK*-mediated innate immunity. Consequently, in addition to causing developmental abnormalities that limit reproductive success, extended early life starvation promotes pathogen resistance later in life.

*C. elegans* L1 arrest and recovery provides a powerful system to investigate the molecular basis of adult consequences of early life starvation. We show that *daf-18/PTEN* suppresses L1 starvation-induced germline tumors and other gonad abnormalities in adults via its well-characterized lipid-phosphatase activity inhibiting PI3K/IIS (Fig 6). In addition, we show that *daf-18* suppresses such starvation-induced abnormalities independently of PI3K/IIS, possibly via its putative protein-phosphatase activity. We show that *lin-35/Rb* also suppresses starvation-induced abnormalities, and that it functions downstream of *daf-18* and independently of IIS. We identified three Hedgehog signaling homologs (*ptr-23/PTCH-related*, *wrt-1/Hh-related*, and *wrt-10/Hh-related*) that promote starvation-induced abnormalities and are antagonized by *lin-35* via DREAM. Along with *tra-1/GLI*, a putative transcriptional effector of Hh-related signaling, these Hh-related genes affect expression of a common set of genes involved in the innate immunity pathway. We demonstrate that innate immunity genes promote development of starvation-induced abnormalities, and that L1 starvation induces immunity, causing increased pathogen resistance in L4 larvae. Induction of bacterial pathogen resistance late in development by early life starvation depends on Hh-related signaling, suggesting a potentially adaptive example of phenotypic plasticity regulated by a novel signaling pathway involving DAF-18/PTEN, LIN-35/Rb, Hh signaling homologs, and an innate immunity response.

### *daf-18/PTEN* and *lin-35/Rb* function independently of insulin/IGF signaling to suppress starvation-induced germline tumors and other gonad abnormalities

PTEN is a potent tumor suppressor that is mutated in multiple advanced cancers at high frequency [67,68], and partial loss of PTEN function, aberrant subcellular localization, and post-translational dysregulation have been linked to tumorigenesis and cancer progression [31]. The sole worm PTEN homolog, *daf-18*, is required to repress cell division and other aspects of postembryonic development during L1 arrest [69], and mutation of *daf-18* causes abnormal gonad development and sterility following relatively brief L1 arrest [19,22]. Furthermore, *daf-18* RNAi during larval development after extended L1 arrest increases the frequency of starvation-induced gonad abnormalities [8], suggesting that DAF-18/PTEN functions as a tumor suppressor beyond L1 arrest. *daf-16/FoxO* mutants have similar L1 arrest-related phenotypes to *daf-18*, but the phenotypes are less severe for *daf-16* (Figs 1 and S1; [6,12,21]). We show that *daf-18* suppression of starvation-induced gonad abnormalities depends in part on PI3K/IIS, but that *daf-18/PTEN* also functions independently of *age-1/PI3K* (Fig 1). PTEN is a dual phosphatase, with lipid and protein-phosphatase activities [20,70]. Protein-phosphatase activity of DAF-18/PTEN has not been demonstrated biochemically, but our results suggest such activity may contribute to suppression of starvation-induced germ cell tumors and other gonad abnormalities. Alternatively, DAF-18 could dephosphorylate a lipid other than PIP3, or it could affect starvation-induced abnormalities non-enzymatically.

The retinoblastoma gene *RB1* was the first tumor suppressor identified [26], and it is mutated in many types of cancer [27,71]. We show that the sole worm RB family homolog, *lin-35/Rb*, represses starvation-induced germ cell tumors (Fig 1), revealing tumor-suppressor activity of LIN-35 for the first time. We recently found that *daf-18/PTEN* requires *lin-35/Rb* to promote starvation resistance during L1 arrest [21], leading us to hypothesize that *daf-18* acts through *lin-35* to suppress development of starvation-induced gonad abnormalities. We show that *lin-35* functions independently of *daf-2/InsR*, that *lin-35* and *daf-18* do not have additive effects on starvation-induced abnormalities, and that *daf-18* affects subcellular localization of LIN-35 (Fig 1). Furthermore, lack of additivity was observed with RNAi, indicating gene function during recovery from starvation. These results suggest that in addition to functioning in a linear pathway to promote starvation survival during L1 arrest [21], *daf-18* and *lin-35* function in a common pathway during larval development to suppress L1 starvation-induced tumors.

### *daf-18/PTEN* and *lin-35/Rb* antagonize Hedgehog-related signaling, which cell-nonautonomously promotes starvation-induced abnormalities

Genes involved in Hedgehog signaling in *Drosophila melanogaster* and mammals are conserved in *C. elegans*, but they are expanded into families of homologs with divergent structures. Among the *C. elegans* Hh-related genes, there are 60 putative ligands, only 10 of which possess the conserved C-terminal auto-processing domain, and 26 putative receptors, 24 of which are classified as “patched-related” (*ptr*) genes [35]. *C. elegans* lacks a Smo homolog, which is repressed by PTCH in mammals and required for canonical Hh signaling, and there is no evidence that the Gli homolog TRA-1 acts as a transcriptional effector for signaling, suggesting that *C. elegans* Hh-related signaling is non-canonical [72]. The phenotypes reported for Hh-related signaling in *C. elegans* are not particularly reminiscent of the roles of Hh signaling in patterning, cell proliferation, and differentiation seen in *D. melanogaster* and mammals [73,74]. However, we show that four Hh-related genes, *ptr-23*, *wrt-1*, *wrt-10*, and *tra-1*, promote starvation-induced germ cell tumors and other gonad abnormalities (Figs 2 and S4), suggesting conserved proto-oncogene function. In addition, we show that *ptr-23*, *wrt-1,* and *wrt-10* are epistatic to *daf-18/PTEN* and *lin-35/Rb* (Fig 2). Epistasis analysis also suggests that *daf-18* and components of the DREAM complex function in a common pathway to suppress starvation-induced abnormalities, and we show that LIN-35 functions with the DREAM complex to repress transcription of *ptr-23*, *wrt-1,* and *wrt-10* (Fig 2). These results suggest that DAF-18 and LIN-35/DREAM antagonize Hh-related signaling through transcriptional repression in larvae that experienced extended L1 arrest. Notably, there is evidence in mammals that loss of *RB1* promotes aberrant cilia formation leading to hypersensitivity to Hh signaling [75], but it is unclear if this relevant to our work given the unciliated epidermis as the apparent major site of action for the Patched receptor-related gene *ptr-23* (Fig 3; see below).

Reporter gene analysis largely corroborated by published scRNA-seq results suggest that *ptr-23*, *wrt-1*, and *wrt-10* are prominently expressed in the epidermis as well as the intestine and other relatively minor sites (Fig 3). Germline expression was not observed, but the multi-copy transgenic arrays analyzed are likely silenced in the germline. Nonetheless, a pair of complementary, RNAi-based approaches suggest that all three genes function in the soma, and not the germline, to promote starvation-induced abnormalities (Fig 3). All three Hh-related genes were required in the epidermis to promote gonad abnormalities, and *wrt-1* was also required in the intestine. In addition, tissue-specific transgenic rescue of *ptr-23*, *wrt-1*, and *wrt-10* mutants suggests that over-expression of each gene in the epidermis or intestine is sufficient to promote starvation-induced abnormalities (Fig 3). Tissue-specific RNAi and transgenic rescue results are consistent with each other. These results are also consistent with the observed expression patterns, though other sites of expression were not tested for function. Epidermal function of Hh-related genes suggests the Hh-related signaling functions cell-nonautonomously to promote germ cell proliferation [6]. We do not know if PTR-23 functions as a receptor for WRT-1 or WRT-10, or if their activity is transduced through TRA-1/GLI as a transcriptional effector. In any case, cell-nonautonomous function suggests that PTR-23, WRT-1, and WRT-10 activity in the epidermis, or possibly intestine, affects at least one other molecule that affects the somatic gonad and/or germ cells to influence their proliferation following extended L1 arrest.

Phenotypic and expression analysis suggests that *ptr-23*, *wrt-1*, and *wrt-10* have much in common, but *wrt-1* stands out in a few ways. *wrt-1* was unique in being necessary and sufficient in both the intestine and epidermis to promote starvation-induced abnormalities (Fig 3). *wrt-1* RNAi during development following extended starvation also affected more genes than *ptr-23* or *wrt-10* (or *tra-1*) (Fig 4). In addition, mutation of *wrt-1* led to the most dramatic decrease in survival on *P. aeruginosa* PA14, which was significantly lower than wild type even in the absence of prior starvation (Fig 5, see below). While *wrt-1* and *wrt-10* both possess a similar *wrt* N-terminal signaling domain, *wrt-1* possesses the conserved C-terminal auto-processing domain, which is absent in the majority of Hh-related putative ligands [76,77]. It is possible that the autocatalytic domain impacts post-translational regulation, secretion, and signaling function, accounting for the more severe effects of loss of *wrt-1*.

### Hedgehog-related signaling mediates activation of innate immunity by early life starvation, promoting developmental abnormalities but increasing pathogen resistance

RNA-seq analysis of adults that had been subjected to extended L1 arrest and then cultured with RNAi targeting *ptr-23*, *wrt-1*, *wrt-10*, or *tra-1* affected a relatively small number of largely overlapping genes (Fig 4). Overlapping effects on gene expression suggest these Hh-related genes do in fact function in a common pathway. Furthermore, genes associated with innate immunity were significantly enriched among genes downregulated by disruption of Hh-related signaling. RNAi of such immunity-associated genes as well as the immunity regulator *pmk-1/p38 MAPK* reduced the frequency of starvation-induced abnormalities, even with hyperactivation of Hh-related signaling (Fig 4), supporting the conclusion that *ptr-23*, *wrt-1*, and *wrt-10* promote transcription of immunity genes. Furthermore, relatively brief (1 d) or extended (8 d) L1 arrest caused upregulation of an immunity marker (*sysm-1*p::GFP) and increased resistance to multiple bacterial pathogens and a eukaryotic intracellular pathogen (Figs 5 and S5). These results suggest that the immunity-associated genes upregulated by Hh-related signaling function to promote immunity against bacterial pathogens in this context.

Connections between *C. elegans* Hh-related signaling, *lin-35/Rb* and innate immunity have been documented [39,40,78,79]. *lin-35* and DREAM have been associated with the intracellular pathogen response (IPR) as a regulator of intergenerational immunity, in addition to roles in chromatin remodeling and RNAi in the context of the antiviral response [78,80,81]. Interestingly, both activation and repression of innate immunity have been observed in different contexts and for different Hh-related genes. Our results suggest that Hh-related signaling, the innate immunity pathway, and starvation-induced gonad abnormalities are causally related, but how an elevated immunity response following extended L1 arrest causes germ cell tumors and other gonad abnormalities is mysterious. Furthermore, the mechanism by which innate immunity is induced during recovery from starvation remains unclear. Our results suggest that the non-pathogenic *E. coli* used to feed the worms induces *sysm-1*p::GFP expression with or without L1 starvation, with a stronger induction in larvae that experienced starvation, and the strongest induction in larvae that experienced extended (8 d) starvation (Fig 5). We suggest that previously starved larvae may have a stronger antigenic response to this commensal food source, rendering larvae subjected to L1 arrest relatively resistant to pathogenic bacteria during late larval development.

L1 arrest enables *C. elegans* larvae to endure long periods of starvation, which is likely ecologically relevant. However, extended L1 arrest has a variety of consequences that are seemingly pathological or maladaptive, including delayed development, reduced adult size, reduced fecundity, reduced progeny size and quality, and germ cell tumors and other gonad abnormalities in adults [6,82,83]. Furthermore, animals that develop gonad abnormalities display the largest reduction in reproductive success [6]. In contrast, we report here that extended L1 arrest increases resistance to a variety of pathogens (Figs 5 and S5), which does not seem pathological and could be adaptive. Increased pathogen resistance could increase fitness following starvation in environments where optimal food sources may not be available. Nonetheless, this potential evolutionary benefit is apparently offset by decanalization of development compromising adult phenotype and reproductive success.

## Materials and methods

***C. elegans* strains (Table 2)**

**Bacterial strains (Table 3)**

### Worm maintenance

Standard culture methods were carried out at 20^o^C on Nematode Growth Media (NGM) agar plates seeded with *E. coli* OP50. For the gonad abnormality assay, reporter quantification, and pathogen resistance assay, plates were seeded with *E. coli* HT115 empty vector bacteria (see below) or other indicated RNAi bacteria. All strains were cultured for at least five generations in well-fed conditions at 20^o^C prior to use in any experiments. All mutants used were backcrossed to wild type N2 for at least 4 generations.

**Table 2:** C. *elegans* strains used in this study.

Strain	Genotype	Description	Source
N2	Wild type	Bristol N2	Paul Sternberg – California Institute of Technology
IC166	*daf-18(ok480)*	Deletion: 956 bp [84]	Ian Chin-Sang - Queen’s University
LRB441	*age-1(m333); daf-18(ok480)*	Double mutant. *age-1 -* Nonsense mutation W641STOP [85]; *daf-18 -* Deletion: 956 bp [84]	Ryan Baugh – Duke University
HRN666	*wrt-10(aus36)*	Deletion: 5 bp [86]	CGC
FX14849	*wrt-1(tm1417)*	Deletion: 648 bp [87]	National BioResource Project (*C. elegans*)
OK3219	*ptr-23(ok3663)*	Deletion: 899 bp [88]	CGC
BR2823	*chn-1(by155)*	Deletion: 989 bp [89]	CGC
RB763	*cwn-1(ok546)*	Deletion: 785 bp [88]	CGC
GG14	*fasn-1(g14)*	Substitution: G1830R [90]	CGC
LRB533	*unc119(ed4); pRB9[wrt-10p::NLS::yfp::unc-54]; pPDMM05[unc-119(+)]*	Multicopy transgene	Generated in this work
LRB551	*unc119(ed4); pRB8[wrt-1p::NLS::yfp::unc-54]; pPDMM05[unc-119(+)]*	Multicopy transgene	Generated in this work
LRB570	*unc119(ed4); pRB10 [ptr-23p::NLS::yfp::unc-54]; pPDMM05[unc-119(+)]*	Multicopy transgene	Generated in this work
NL2550	*ppw-1(pk2505)*	Single base deletion, nonsense mutation [52]	CGC
NL2098	*rrf-1(pk1417)*	Deletion: 1203 bp [51]	CGC
WM27	*rde-1(ne219)*	Substitution: E414K [54]	CGC
NR222	*rde-1(ne219); kzIs9 [(pKK1260) lin-26p::NLS::gfp + (pKK1253) lin-26p::rde-1 + rol-6(su1006)]*	Tissue-specific rescue. *rde-1 -* Substitution: E414K [54]; kzIs9 - Multicopy transgene [55]	CGC
VP303	*rde-1(ne219) V; kbIs7 [nhx-2p::rde-1 + rol-6(su1006)]*	Tissue-specific rescue. *rde-1 -* Substitution: E414K [54]; *kbIs7* - Multicopy transgene [56]	CGC
AMJ345	*jamSi2 [mex-5p::rde-1(+)]; rde-1(ne219)*	Tissue-specific rescue. *rde-1 -* Substitution: E414K [54]; Single-copy insertion [57]	CGC
LRB622	*unc-199(ed4); wrt-1 (tm1417); dukEx148[wrt-1p::wrt-1::unc-54 3’ UTR + unc-119(+)]*	Tissue-specific rescue: *wrt-1* - Deletion: 648 bp [87] Multicopy transgene (This work)	Generated in this work
LRB623	*unc-199(ed4); wrt-10 (aus36); dukEx151[col-12p::wrt-10::unc-54 3’ UTR + unc-119(+)]*	Tissue-specific rescue: *wrt-*10 - Deletion: 5 bp [86]; Multicopy transgene (This work)	Generated in this work
LRB624	*unc-199(ed4); ptr-23 (ok3663); dukEx152[col-12p::ptr-23::unc-54 3’ UTR + unc-119(+)]*	Tissue-specific rescue: *ptr-23* - Deletion: 899 bp [88]; Multicopy transgene (This work)	Generated in this work
LRB625	*unc-199(ed4); wrt-10 (aus36); dukEx153[ges-1p::wrt-10::unc-54 3’ UTR + unc-119(+)]*	Tissue-specific rescue: *wrt-*10 - Deletion: 5 bp [86]; Multicopy transgene (This work)	Generated in this work
LRB626	*unc-199(ed4); ptr-23 (ok3663); dukEx154[ges-1p::ptr-23::unc-54 3’ UTR + unc-119(+)]*	Tissue-specific rescue: *ptr-23* - Deletion: 899 bp [88]; Multicopy transgene (This work)	Generated in this work
LRB627	*unc-199(ed4); wrt-1 (tm1417); dukEx150[col-12p::wrt-1::unc-54 3’ UTR + unc-119(+)]*	Tissue-specific rescue: *wrt-1* - Deletion: 648 bp [87]; Multicopy transgene (This work)	Generated in this work
LRB636	*unc-199(ed4); ptr-23 (ok3663); dukEx155[ptr-23p::ptr-23::unc-54 3’ UTR + unc-119(+)]*	Tissue-specific rescue: *ptr-23* - Deletion: 899 bp [88]; Multicopy transgene (This work)	Generated in this work
LRB637	*unc-199(ed4); wrt-10 (aus36); dukEx149[wrt-10p::wrt-10::unc-54 3’ UTR + unc-119(+)]*	Tissue-specific rescue: *wrt-*10 - Deletion: 5 bp [86]; Multicopy transgene (This work)	Generated in this work
LRB643	*unc-199(ed4); wrt-1 (tm1417); dukEx156[ges-1p::wrt-1::unc-54 3’ UTR + unc-119(+)]*	Tissue-specific rescue: *wrt-1* - Deletion: 648 bp [87]; Multicopy transgene (This work)	Generated in this work
LRB644	*unc-199(ed4); wrt-10 (aus36); dukEx157[mex-5p::wrt-10::nos-2 3’ UTR + unc-119(+)]*	Tissue-specific rescue: *wrt-*10 - Deletion: 5 bp [86]; Multicopy transgene (This work)	Generated in this work
LRB645	*unc-199(ed4); wrt-1 (tm1417); dukEx158 [mex-5p::wrt-1::nos-2 3’ UTR + unc-119(+)]*	Tissue-specific rescue: *wrt-1* - Deletion: 648 bp [87]; Multicopy transgene (This work)	Generated in this work
LRB646	*unc-199(ed4); ptr-23 (ok3663); dukEx159[mex-5p::ptr-23::nos-*2 *3’ UTR + unc-119(+)]*	Tissue-specific rescue: *ptr-23* - Deletion: 899 bp [88]; Multicopy transgene (This work)	Generated in this work
AU78	*agIs219 [T24B8.5sysm-1p::gfp::unc-54 3’ UTR + ttx-3p::gfp::unc-54 3’ UTR]*	Single copy insertion [66]	CGC
KU25	*pmk-1(km25)*	Deletion: 375 bp [91]	CGC
NK1228	*qyIs288 [daf-16p:: > gfp::DAF-16 + unc-119(+)]; daf-16(mu86); unc-119(ed4)*	*qyIs288* – multicopy transgene [92]. *daf-16(mu86)* - 10980 bp deletion [93]	Adam Schindler, David Sherwood – Duke University
SPC167	*dvIs19 [(pAF15) [gst-4p::gfp::NLS]; skn-1(lax120)*	*dvIs19 [(pAF15)gst-4p::gfp::NLS]* – single copy insertion [94] *skn-1(lax120)* – Substitution S245L [95]	CGC
AWR58	*lin-35(kea7[lin-35p*::degron::*gfp*::*lin-35*]); *keaSi10[rpl-28p*::TIR1::*mRuby*::*unc-54* 3’UTR + Cbr-unc-119(+)]	*lin-35(kea7): degron/gfp* insertion *keaSi10:* single copy insertion [78]	CGC
ERT356	*pals-22(jy1)*	Substitution: R51STOP [96]	Aaron Reinke – University of Toronto

**Table 3:** Bacterial strains used in this study.

Species	Strain	Source
*Escherichia coli*	OP50	CGC
*Pseudomonas aeruginosa*	PA14	Joel Myer – Duke University
*Pseudomonas aeruginosa*	SM381 - pRhlA-mNeonGreen	Bonnie Bassler – Princeton University
*Salmonella enterica*	SL1344	Joel Myer – Duke University
*Escherichia coli*	HT115 – L4440 empty vector	Coleen Murphy – Princeton University
*Escherichia coli*	HT115 – *gfp* RNAi	Craig Hunter – Harvard University
*Escherichia coli*	HT115 – *daf-18* RNAi	Open Biosystems – David Sherwood - Duke University
*Escherichia coli*	HT115 – *lin-35* RNAi	Open Biosystems – David Sherwood - Duke University
*Escherichia coli*	HT115 – *daf-2* RNAi	Colleen Murphy – Princeton University
*Escherichia coli*	HT115 – *ptr-23* RNAi	Open Biosystems - David Sherwood - Duke University
*Escherichia coli*	HT115 – *wrt-1* RNAi	Open Biosystems - David Sherwood - Duke University
*Escherichia coli*	HT115 – *wrt-10* RNAi	Open Biosystems - David Sherwood - Duke University
*Escherichia coli*	HT115 – *tra-1* RNAi	Open Biosystems - David Sherwood - Duke University
*Escherichia coli*	HT115 – *dod-24* RNAi	Open Biosystems - David Sherwood - Duke University
*Escherichia coli*	HT115 - F55G11.8 RNAi	Open Biosystems - David Sherwood - Duke University
*Escherichia coli*	HT115 - F56A4.2 RNAi	Open Biosystems - David Sherwood - Duke University
*Escherichia coli*	HT115 – *oac-6* RNAi	Open Biosystems - David Sherwood - Duke University
*Escherichia coli*	HT115 - Y47H10A.5 RNAi	Open Biosystems - David Sherwood - Duke University
*Escherichia coli*	HT115 - C18H9.6 RNAi	Open Biosystems - David Sherwood - Duke University
*Escherichia coli*	HT115 – *ugt-44* RNAi	Ahringer Library – David Sherwood – Duke University
*Escherichia coli*	HT115 – *irg-5* RNAi	Ahringer Library – David Sherwood – Duke University
*Escherichia coli*	HT115 – *cpr-3* RNAi	Ahringer Library – David Sherwood – Duke University
*Escherichia coli*	HT115 - K08D8.5 RNAi	Ahringer Library – David Sherwood – Duke University
*Escherichia coli*	HT115 – *asp-14* RNAi	Ahringer Library – David Sherwood – Duke University
*Escherichia coli*	HT115 – *clc-19* RNAi	Ahringer Library – David Sherwood – Duke University
*Escherichia coli*	HT115 – *efl-1* RNAi	Open Biosystems - David Sherwood - Duke University
*Escherichia coli*	HT115 – *lin-9* RNAi	Open Biosystems - David Sherwood - Duke University
*Nematocida parisii*	ERTm1	Aaron Reinke – University of Toronto

### Hypochlorite treatment and starvation culture preparation

For starvation cultures used for scoring gonad abnormalities, sterile, synchronized embryos were obtained by hypochlorite treatment of adults on the first day of egg laying. Eight adults on the first day of egg laying were transferred to a 10 cm NGM plate seeded with *E. coli* OP50. After 72 hours at 20^o^C, progeny (in their first day of egg laying) were washed from the plates with S-basal medium and hypochlorite-treated to obtain embryos. For all other assays, seven L4 larvae were transferred and grown for 96 hours prior to hypochlorite treatment [97]. Unless otherwise noted (Fig 1A), embryos were transferred to virgin S-basal (without ethanol and cholesterol) and maintained at 20^o^C on a tissue-culture roller drum at a density of 1 animal per µL to hatch and enter L1 arrest.

### RNA interference

*E. coli* HT115 bacteria carrying a plasmid expressing double-stranded RNA for the indicated gene (or the empty vector control L4440) were used in all treatments. All RNAi bacteria used are listed above. Glycerol stocks were stored at -80^o^C. Frozen stocks were streaked on Luria Bertani (LB) plates with carbenicillin (Carb; 100 mg/ml) and tetracycline (Tet; 12.5 mg/ml) and grown overnight at 37^o^C. A single colony was picked into 1 mL LB + Carb (50 mg/ml) + Tet (12.5 mg/ml) and grown overnight at 37^o^C and 250 rpm. This culture was used to inoculate 5–20 mL of Terrific Broth (TB) with Carb (50 mg/mL) and grown overnight at 37^o^C and 300 rpm. Turbid cultures were centrifuged for 10 minutes at 4000 rpm and the supernatant was removed. Bacteria were resuspended in S-complete + 20% glycerol at a 4:1 ratio by mass. 100 µL aliquots were frozen and stored at -80^o^C. 15 µL from one of these aliquots was used to inoculate 6 cm NGM + Carb (50 mg/ml) + IPTG (1 mM) plates and grown overnight at room temperature before use in experiments. If two RNAi treatments were used in combination, 10 μL from each aliquot were mixed then 15 μL from this mixture was plated and spread. To control for dilution, single RNAi controls were mixed with *gfp* RNAi bacteria, and *gfp* RNAi bacteria was used as a negative control.

### Scoring gonad abnormalities

For extended starvation conditions, L1 larvae were typically arrested in virgin S-basal for 8 days after hypochlorite treatment, but in limited cases they were arrested for 4 days and/or 0.1% ethanol was added to S-basal (Fig 1A). Two types of control conditions were used, as indicated: 1) Embryos were allowed to hatch and arrest overnight for synchronization, approximately 18 hours after hypochlorite treatment (“1 day L1 arrest”), or 2) embryos were plated directly with food so they hatch and proceed directly to postembryonic development without entering L1 arrest (“Unstarved”). 150 arrested L1 larvae or unhatched embryos were transferred to 6 cm NGM + Carbenicillin + IPTG plates seeded with *E. coli* HT115 expressing an empty vector RNAi plasmid (L4440) or indicated RNAi treatment. Worms were grown to early adulthood (approximately 60 hours for animals starved for 18 hours, 68 hours for animals starved for 4 days, and approximately 72 hours for animals starved for 8 days or unhatched embryos) then washed with S-basal and anesthetized with levamisole (10 mM). Paralyzed adults were transferred to 4% noble agar pads on microscope slides and viewed at 200X total magnification using differential interference contrast/Nomarski (DIC) microscopy on a Zeiss AxioImager compound microscope. Fifty individuals were scored for the presence of proximal germ cell tumors, uterine masses, or other obvious gonad abnormalities (S1A and S1B Fig [9]). Proximal germ cell tumors present as large masses near the vulva/proximal gonad with visible germ cell nuclei (similar to the mitotic distal gonad), and uterine masses are characterized as large irregular tissues in the uterus without visible germ cell nuclei (differentiated) often resulting in extruded vulvae. For statistical analysis, Bartlett’s test was used to determine homogeneity of variance, and if non-significant, variance was pooled for subsequent analysis. Two-tailed, unpaired t-tests were used for pairwise comparisons.

### Scoring LIN-35 subcellular localization

Starvation cultures were prepared for GFP::LIN-35 in wild type and *daf-18(ok480)* backgrounds as described above for scoring gonad abnormalities. Following 1 or 8 days of starvation, 500 L1 larvae were transferred to empty vector *E. coli* HT115. After 48 hours at 20^o^C, larvae were washed with 1 mL of S-basal then transferred to 1.5 mL Eppendorf tubes and centrifuged at 3000 rpm for 1 minute. 4 µL was transferred from the pellet to a 4% noble agar pad and covered with a coverslip. Larvae were visualized at 200x total magnification on a Zeiss AxioImager compound microscope. Subcellular localization was scored with four categories ranging from completely nuclear to completely cytoplasmic. A Cochran-Mantel-Haenszel Chi-squared test was used to perform pairwise comparisons between genotypes and conditions for the distribution of GFP::LIN-35 subcellular localization categories.

### Plasmid design and cloning

Gibson assembly was used for making new plasmids. Q5 high-fidelity polymerase PCR (NEB M0491) was used to amplify plasmid fragments from plasmid DNA and promoter sequences from wild-type genomic DNA (see S6 Table for PCR primer sequences). DNA sequences for design were obtained from WormBase [98]. Hedgehog-related gene coding sequences were obtained from genomic DNA, *yfp* reporter genes with the plasmid backbone were sub-cloned from pPD132.112 (Andy Fire Lab Vector Kit), and the pNL213, pAS10, and pGC550 plasmids, which include promoters and 3’ UTRs for intestine, epidermis, and germline expression, respectively, were used as vectors. PCR fragments were gel purified (Zymo Research D4001). Purified DNA was used for Gibson assembly based on the kit manufacturer instructions, and the resulting products were used for bacterial transformation using competent cells from the same kit (NEB E5510S). Plasmid DNA was extracted from individual colonies (Zymo Research D4016), and plasmids were sequenced to confirm their structure.

### Plasmid microinjection

Microinjection was used to generate all multicopy transcriptional reporter lines and tissue-specific rescue lines used in this work. Worms carrying the *unc-119(ed4)* mutation were used for all injections. Young adults (not yet gravid) or late L4 larvae were picked onto unseeded (lacking bacterial food) NGM plates. A paintbrush and halocarbon oil were used to transfer worms onto a desiccated 2% noble agar pad on a 45 mm x 50 mm cover glass. Plasmids were injected into the distal gonad at a concentration of 100 ng/μl. An *unc-119* rescue plasmid (pPDMM051) was used as an injection marker at a concentration of 50 ng/μl. Injected animals were transferred to 2 cm NGM plates with seeded with *E. coli* OP50, and transformants were identified among progeny by *unc-119* rescue (wild type morphology and motility). Transformed progeny were singled to new plates, and stable lines were genotyped for validation by confirming GFP expression or PCR targeting the promoter-coding sequence fusion in the transgene.

### Compound microscopy imaging of reporter genes

Well-fed L4 larvae were transferred to 2 μL of 10 mM Levamisole on a 4% noble agar pad on a microscope slide and covered with a coverslip. Worms were imaged at 200x (AU78) or 400X (LRB533, LRB551, LRB570) total magnification using a Zeiss AxioImager compound microscope with an AxioCam 506 Mono camera. FIJI and Adobe Illustrator were used for miscellaneous editing, cropping, and stitching of images.

### RNA-seq sample collection

Starvation cultures were prepared as for scoring gonad abnormalities as described above. Following 8 days of starvation, 150 L1 larvae were plated onto empty vector, *ptr-23, tra-1, wrt-1,* and *wrt-10* RNAi. After 68 hours, adults were washed with S-basal from plates then centrifuged at 3000 rpm for 1 minute. Worms were washed in 10 mL of S-basal three additional times, and a final volume of 100 μL was transferred to a 1.5 mL Eppendorf tube. Samples were frozen in liquid nitrogen and stored at -80^o^C.

### RT-qPCR sample collection

Starvation cultures were prepared as described above for scoring gonad abnormalities. Following 1 or 8 days of starvation, 20,000 arrested L1 larvae were centrifuged, and the supernatant was aspirated to a final volume of 100 μL then transferred to a 1.5 mL Eppendorf tube and frozen in liquid nitrogen. Additionally, 10,000 larvae were transferred to empty vector RNAi *E. coli* HT115 then collected after 24 hours, 500 larvae were collected after 48 hours, and 150 adults were collected after 72 hours at 20^o^C. Wild type and mutants collected in Fig 2I were collected frozen after 48 hours of recovery.

### RNA extraction

100 μL of acid-washed sand and 1 mL of TRIzol were added to frozen worm pellets, and the tubes were vortexed vigorously for 10 minutes to homogenize samples. 200 μL of phenol-chloroform was added, and the mixture was vigorously vortexed for an additional 3 minutes. Tubes were centrifuged for 3 minutes at 21,000 rpm. A p200 micropipette was used to extract the aqueous layer, which was transferred to a new 1.5 mL Eppendorf tube and mixed with 500 μL of isopropanol. The mixture was incubated for 8 minutes at room temperature followed by 2 minutes on ice. Tubes were subsequently centrifuged for 10 minutes at 21,000 rpm. RNA pellets were washed with 75% ethanol twice, after which they were allowed to air dry prior to resuspension in 10 μL of water. RNA quality and quantity were analyzed on NanoDrop and Qubit instruments, respectively.

### Reverse transcription

5 µg of total RNA was mixed with 150 ng of random hexamers and 1 µg of 10 mM dNTP mix then incubated at 65^o^C for 5 minutes. 2 µL 50 mM MgCl_2_, 2 µL 0.1 M DTT, and 1 µL RNAaseOUT was added to a 1X RT buffer with 50 units of Superscript IV RT then added to the previous RNA mixture. Samples were incubated at 20^o^C for 10 minutes, 42^o^C for 50 minutes, then heat inactivated at 70^o^C for 15 minutes before long-term storage at -20^o^C.

### RT-qPCR

One-tenth of the cDNA (2 μL) from each RT reaction was used as a template with standard Taq Polymerase conditions plus SYBR green (S7563) with primers designed for the genes of interest in a 25 µL reaction. Plates were loaded into Roche LightCycler 96 machine and amplified for 40 cycles. Cq values were estimated and *tct-1* was used as a housekeeping gene for normalization. Two technical replicates were performed for three biological replicates (distinct populations) then averaged. Pairwise t-tests were used to compare the mean normalized Cq values.

### RNA-seq Library preparation

RNA-seq libraries were prepared for sequencing using the NEBNext Ultra II RNA Library Prep Kit for Illumina (New England Biolabs #E7775) starting with 1 μg of total RNA per sample as input and seven cycles of PCR. Individually barcoded libraries were pooled and sequenced on the NovaSeq 6000 S-Prime flow cell to obtain 50 bp paired-end reads.

### RNA-seq analysis

Bowtie was used for mapping reads to the WS273 *C. elegans* genome and HTSeq was used for counting reads and generating count tables using a Linux command line on Ubuntu. EdgeR was used to estimate dispersion and perform an exact test for differential expression analysis on R Windows. A false discovery rate < 0.1 was used as a significance threshold for pairwise differential gene expression. A generalized linear model with a p-value cutoff < 0.05 was used for identifying differentially expressed genes across all treatments for clustering by log_2_ fold-change compared to the empty vector control. WormCat [61] was used for gene set enrichment analysis.

### Bacterial pathogen survival

Fast-killing or slow-killing NGM plates [64] were seeded with 15 μL of *P. aeruginosa* PA14 or *S. enterica* SL1344 bacteria, which was spread to cover the entire plate surface to prevent pathogen avoidance, and incubated overnight at 37^o^C. Synchronized populations of unstarved L1s and L1s starved for 1 or 8 days were prepared as described above. 500 L1 larvae/embryos were plated onto *E. coli* HT115 bacteria and cultured for 56 hours for embryos, 48 hours for larvae starved for 1 day, and 56 hours for larvae starved for 8 days at 20^o^C. Fifty L4 larvae were picked onto pathogen plates. Survival was scored by observing movement. If worms were unresponsive to gentle prodding with a pick, they were removed from the plate and scored as dead. Worms that died from other causes, such as crawling onto the side of the plates, were censored. Survival was scored every 2 hours for the first 6 hours, 18 hours later (24 hours total), 6 hours later (30 hours total), 18 hours later, 6 hours later, and so on until no live worms remained. Oasis 2 [99] was used for statistical analysis and the log-rank test was used for pairwise comparisons between strains.

### Microsporidia infection

1000 synchronized L1s starved for 1 or 8 days were concentrated in 100 μL and mixed with 400 μL of 10X OP50 and 0.72 million *N. parisii* ERTm1 spores. The mixture was spread to cover the surface of a 6 cm NGM plate. After incubation at 21°C for 72 h, the worms were fixed in acetone, stained with Direct Yellow 96 (DY96) and resuspended in EverBrite Mounting Medium with DAPI [78]. Worms were visualized using an Axio Imager 2 (Zeiss) and scored based on the presence of intracellular DY96-stained spores.

### Automated imaging and quantitative analysis of reporter gene expression

Synchronized populations of unstarved L1s and L1s starved for 1 or 8 days were prepared as described above. 500 L1 larvae/embryos were plated onto empty vector *E. coli* HT115, or the indicated RNAi treatment, and cultured for 56 hours for embryos, 48 hours for L1 larvae starved for 1 day, and 56 hours for L1 larvae starved for 8 days, or the indicated duration, at 20^o^C. Worms were washed from plates with virgin S-basal and briefly centrifuged (15 seconds at 3000 rpm). Worms were resuspended in 390 µL of 50 μM sodium azide and transferred to a 96-well plate. Plates were imaged on an ImageXpress Nano automated imager at 100X total magnification. Sites for the same well were stitched and objects resulting from automatic segmentation were analyzed to measure average fluorescent intensity. Objects were filtered by size (5 µm < width < 40 µm and 10000 < pixels < 75000), and background intensity was subtracted. Manual screening of objects also removed any instances of multiple animals or other debris. A linear mixed-effects model (ex: lme(intensity~strain random = ~1|replicate, data = imager) was used to fit average intensity per individual as the response variable, strain as the fixed effect, and replicate as the random effect for pairwise comparisons.

### Starvation survival

Starvation cultures were prepared as described above. After 1 day, 100 μL of the starvation culture was plated onto a 2 cm NGM plate seeded with *E. coli* OP50, and the number of plated L1 larvae was counted. After 48 hours at 20^o^C, the number of live larvae (exhibited growth or were still actively moving on plate surface) was counted, and the proportion of survivors was determined by dividing the number plated by the number of survivors. Survival was scored every 24 hours until there were no survivors. Quasi-binomial logistic regression, with the frequency of survivors as the response variable and the duration of starvation as the explanatory variable, was used for curve fitting and to estimate half-lives for each biological replicate. Bartlett’s test was used to determine if variance in replicate half-lives was homogeneous across genotypes, and if it was then variance was pooled across genotypes for subsequent analysis. Two-tailed, unpaired t-tests on replicate half-lives were used for pairwise comparisons between genotypes.

### Quantification of intestinal bacterial loads

Synchronized populations of unstarved L1s and L1s starved for 8 days were prepared as described above. 500 L1 larvae/embryos were plated onto *E. coli* HT115 bacteria on 10 cm plates and cultured for 56 hours at 20^o^C. Fifty L4 larvae were transferred to *P. aeruginosa* SM381 on fast-killing plates prepared as for pathogen survival. After two hours, worms were transferred to *E. coli* OP50 for 20 minutes for three times to remove any *P. aeruginosa* on the surface of worms. 10 worms were transferred to 50 μL PBS plus 0.1% Triton in a 1.5 mL Eppendorf tube and ground with a pestle to release intestinal bacterial contents into the lysate. The lysates were diluted 10-fold then spread on LB media and incubated overnight at 37^o^C. Fluorescent colonies were counted across a 1/8 section (counts ranged from 500-1000 CFUs) and total CFUs per individual were estimated.

### Pumping rate quantification

Pumping rate was measured as described in [100]. Synchronized populations of unstarved L1s and L1s starved for 8 days were prepared as described above. 500 L1 larvae/embryos were plated onto *E. coli* HT115 bacteria and cultured for 56 hours at 20^o^C. Fifty L4 larvae were transferred to *P. aeruginosa* PA14 on fast-killing plates prepared as for pathogen survival. After two hours, worms on PA14 and HT115 were visualized under a Zeiss Discovery.V20 SteREO microscope and pharyngeal pumps were counted for 30 seconds. Three counts were conducted per animal then averaged for a total of 10 animals per biological replicate of a condition.

### Defecation rate quantification

Defecation cycle time was measured as described in [100]. Synchronized populations of unstarved L1s and L1s starved for 8 days were prepared as described above. 500 L1 larvae/embryos were plated onto *E. coli* HT115 bacteria on 10 cm plates and cultured for 56 hours at 20^o^C. Fifty L4 larvae were transferred to *P. aeruginosa* PA14 on fast-killing plates prepared as for pathogen survival. After two hours, worms on PA14 and HT115 were visualized on a Zeiss Discovery.V20 SteREO microscope to visualize the rectal muscles. After an initial contraction of the rectal muscles and intestinal expulsion, the duration of the defecation cycle was measured. Three counts were conducted per animal then averaged with a total of 10 animals per biological replicate of a condition.

## Supporting information

S1 Fig IIS effector genes do not account for the relatively strong effect of *daf-18/PTEN* on starvation-induced gonad abnormalities, and *daf-18* affects GFP::LIN-35 subcellular localization (related to Fig 1). A) Representative image of a healthy wild-type adult without obvious gonad abnormalities after extended L1 arrest. B) Representative image of a wild-type adult with the most common gonad abnormalities observed after extended L1 arrest, including a differentiated uterine mass and a proximal germ cell tumor. A, B) Images of following extended L1 arrest (8 days) were taken at 400x total magnification 72 hr after plating with food at 20°C. Images were taken with differential interference contrast (DIC). FIJI and Adobe Illustrator were used for adjusting brightness/contrast and stitching images. Relevant tissues, organs, and abnormalities are outlined or indicated with arrowheads and labels. C, E) Wild-type larvae were starved during L1 arrest for 8 days then recovered with RNAi bacteria targeting the indicated genes and scored for gonad abnormalities. D) Wild type, *daf-16(mu86);* a *daf-16* over-expression (OE) strain (*qyIs288 [daf-16p::gfp::DAF-16]*), and a *skn-1* gain-of-function (GoF) strain (*skn-1(lax120)*) were starved for 8 days then recovered on empty vector RNAi bacteria and scored for gonad abnormalities. F) Wild type larvae were starved for the indicated duration then recovered on the indicated RNAi bacteria and scored for gonad abnormalities. C-F) Each dot represents a biological replicate including ~50 individuals (see S1 Table for summary statistics). Horizontal bars represent the mean across replicates. Asterisks indicate statistically significant differences between RNAi treatments and *gfp* control (C), between mutants and wild type (B) (no statistical significance in E or F). **** P < 0.0001; unpaired, two-tailed t-test. H) Wild-type and *daf-18(ok480)* worms expressing GFP::LIN-35 were starved for 1 or 4 days during L1 arrest with 0.1% ethanol, recovered on *E. coli* HT115 plates for 48 hr, and imaged.
(TIF)

S2 Fig *ptr-23*, *wrt-1*, and *wrt-10* mutants do not have increased L1 starvation survival, and the *chn-1* mutant does not enhance abnormalities without extended L1 arrest (related to Fig 2). A) L1 starvation survival was scored for the indicated genotypes in three biological replicates. ~ 100 individuals (median = 92, range = 51–186) were scored per time point in each genotype. Curves were fit to each genotype with logistic regression and were used to calculate half-lives per replicate for each genotype, and an unpaired two-tailed t-test was used for pairwise comparisons of half-lives between each mutant and wild type. No significant differences in survival were observed. See S2 Table for summary statistics. B) Wild type and *chn-1(by155)* larvae were starved for the indicated duration then recovered on empty vector RNAi and scored for gonad abnormalities. Each dot represents a biological replicate with ~50 individuals (see S1 Table for summary statistics). Horizontal bars represent the mean across replicates. An unpaired, two-tailed t-test was used for pairwise comparisons between genotypes with the same duration of starvation. No significant differences in abnormality frequency were observed.
(TIF)

S3 Fig Overexpression of *wrt-10*, *ptr-23*, and *wrt-1* does not cause gonad abnormalities without extended L1 arrest (related to Fig 3). Wild type and *ptr-23(ok3663), wrt-1(tm1417),* and *wrt-10(au36)* mutants rescued with a multicopy transgene with tissue-specific expression in the intestine (*ges-1p*; see Fig 3D) were starved for the indicated duration then recovered on empty vector RNAi bacteria and scored for gonad abnormalities. Each dot represents a biological replicate with ~50 individuals (see S1 Table for summary statistics). Horizontal bars represent the mean across replicates. Unpaired, two-tailed t-test between each mutant and wild type with the same duration of starvation revealed no significant differences in abnormality frequency.
(TIF)

S4 Fig *tra-1/GLI* knockdown suppresses starvation-induced gonad abnormalities (related to Fig 4). Wild type L1 larvae were starved for 8 days then recovered on empty vector RNAi bacteria or *tra-1* RNAi and scored for gonad abnormalities on the first day of egg laying. Each dot represents a biological replicate with ~50 individuals (see S1 Table for summary statistics). Horizontal bars represent the mean across replicates. * P < 0.05; unpaired, two-tailed t-test.
(TIF)

S5 Fig Starvation during L1 arrest activates innate immunity later in life (related to Fig 5). A, B) Wild-type L1 larvae were starved for the indicated duration, recovered on plates with *E. coli* HT115 bacteria for 48 hr, and L4 larvae were transferred to *P. aeruginosa* PA14 “slow-killing” plates (A) or *S. enterica* plates (B), and survival was assayed. Three biological replicates were performed with ~50 individuals each (see S1 Table for summary statistics). Mean survival at each timepoint is plotted as a line with survival of each replicate at each timepoint included as dots. Asterisks indicate significance between the indicated starvation durations and unstarved. The log-rank test was used for pairwise comparisons; C) Wild-type, *wrt-1(tm1417), wrt-10(au36)* and *pals-22(jy1)* L1 larvae were starved for the indicated duration and recovered on plates with *E. coli* OP50 bacteria and *N. parisii* spores for 72 hr at 21°C, stained with DY96, and the proportion of individuals with intracellular microsporidia was determined by microscopy. The *pals-22* mutant is included as a control, since it has previously been shown to be resistant [78]. D) Images of *sysm-1*p::GFP expression with a total magnification of 200x were taken of a random population of L4 larvae starved for the indicated duration (as in Fig 5B). To ensure matching stages, worms starved for 0 hours were allowed an additional 8 hr to account for embryogenesis, and worms starved for 8 d were also allowed an additional 8 hr to account for developmental delay following extended starvation. Corresponding DIC images are also provided. E, F) Wild-type, *pmk-1(km25)*, *ptr-23(ok3663)*, *wrt-1(tm1417)*, and *wrt-10(aus36)* L1 larvae were starved for 8 days or plated directly after hypochlorite treatment onto empty vector RNAi bacteria (HT115) plates. After 48 hours of feeding, larvae were transferred to PA14 “fast killing” plates or kept on empty vector RNAi then assayed for pumping rate (E) or defecation cycle time (F). Each dot represents the mean from 10 individuals in a single biological replicate. Horizontal bars represent the mean across replicates within the same conditions. A two-tailed pairwise t-test was used for all statistical comparisons. Bars and asterisks indicate statistical significance. A-C, E, F) * P < 0.05, ** P < 0.01, *** P < 0.001.
(TIF)

S1 Table Gonad abnormalities summary statistics. Average number of animals scored and mean frequency abnormal is reported for all experiments. Corresponding figure panels are indicated.
(XLSX)

S2 Table Starvation survival. Mean number of L1 larvae plated from starvation cultures, maximum and minimum number of L1 larvae plated, and half life is reported for all biological replicates for each experimental condition in S2 Fig. Source data is also provided detailing complete scoring.
(XLSX)

S3 Table RNA-seq. Experimental details including sample collection, analysis parameters, and mapping efficiency are reported. Counts per million are reported for all mapped genes for each biological replicate in all experimental conditions. Fold changes, counts per million, p-values and false discovery rates are reported from the output of a generalized linear model. Fold changes, counts per million, p-values and false discovery rates are also reported from the output of an exact test for all comparisons between Hh-related gene RNAi-treated larvae and negative controls recovered on empty vector RNAi. Additionally, an exact test was performed pooling all biological replicates from the four Hh-related RNAi conditions and fold changes, counts per million, p-values and false discovery rates are reported. Finally, the complete gene enrichment output for all differentially expressed genes identified from the exact test on pooling all Hh-related RNAi treatment conditions from WormCat is provided.
(XLSX)

S4 Table Pathogen survival. Source data for all *Pseudomonas aeruginosa, Salmonella enterica,* and *Nematocida parisii* survival assay scoring is provided.
(XLSX)

S5 Table Reporter quantification summary statistics. The total number of individuals quantified and mean pixel intensity is reported for all experimental conditions for all image quantification assays. Corresponding figure panels are indicated.
(XLSX)

S6 Table Primers and plasmids. Primer sequences and DNA templates are provided for all plasmids generated in this study.
(XLSX)

S7 Table Raw data. Source data is provided for all gonad abnormality scoring, feeding, defecation, intestinal bacterial load quantification, RT-qPCR, and image quantification assays.
(XLSX)
